# CardiSort: a convolutional neural network for cross vendor automated sorting of cardiac MR images

**DOI:** 10.1007/s00330-022-08724-4

**Published:** 2022-04-04

**Authors:** Ruth P. Lim, Stefan Kachel, Adriana D. M. Villa, Leighton Kearney, Nuno Bettencourt, Alistair A. Young, Amedeo Chiribiri, Cian M. Scannell

**Affiliations:** 1 Austin Health, Melbourne, Australia; 2 Departments of Radiology, The University of Melbourne, Melbourne, Australia; 3 Department of Surgery (Austin), The University of Melbourne, Melbourne, Australia; 4 Department of Radiology, Columbia University, New York, USA; 5 School of Biomedical Engineering and Imaging Sciences, Kings College London, London, UK; 6 I-MED Radiology, Melbourne, Australia; 7 Cardiovascular R & D Unit, University of Porto, Porto, Portugal

**Keywords:** Magnetic resonance imaging, Deep learning, Workflow, Heart, Humans

## Abstract

**Objectives:**

To develop an image-based automatic deep learning method to classify cardiac MR images by sequence type and imaging plane for improved clinical post-processing efficiency.

**Methods:**

Multivendor cardiac MRI studies were retrospectively collected from 4 centres and 3 vendors. A two-head convolutional neural network (‘CardiSort’) was trained to classify 35 sequences by imaging sequence (*n* = 17) and plane (*n* = 10). Single vendor training (SVT) on single-centre images (*n* = 234 patients) and multivendor training (MVT) with multicentre images (*n* = 434 patients, 3 centres) were performed. Model accuracy and F1 scores on a hold-out test set were calculated, with ground truth labels by an expert radiologist. External validation of MVT (MVT_external_) was performed on data from 3 previously unseen magnet systems from 2 vendors (*n* = 80 patients).

**Results:**

Model sequence/plane/overall accuracy and F1-scores were 85.2%/93.2%/81.8% and 0.82 for SVT and 96.1%/97.9%/94.3% and 0.94 MVT on the hold-out test set. MVT_external_ yielded sequence/plane/combined accuracy and F1-scores of 92.7%/93.0%/86.6% and 0.86. There was high accuracy for common sequences and conventional cardiac planes. Poor accuracy was observed for underrepresented classes and sequences where there was greater variability in acquisition parameters across centres, such as perfusion imaging.

**Conclusions:**

A deep learning network was developed on multivendor data to classify MRI studies into component sequences and planes, with external validation. With refinement, it has potential to improve workflow by enabling automated sequence selection, an important first step in completely automated post-processing pipelines.

**Key Points:**

*• Deep learning can be applied for consistent and efficient classification of cardiac MR image types.*

*• A multicentre, multivendor study using a deep learning algorithm (CardiSort) showed high classification accuracy on a hold-out test set with good generalisation to images from previously unseen magnet systems.*

*• CardiSort has potential to improve clinical workflows, as a vital first step in developing fully automated post-processing pipelines.*

**Supplementary Information:**

The online version contains supplementary material available at 10.1007/s00330-022-08724-4.

## Introduction

Cardiac magnetic resonance studies are commonly performed for comprehensive anatomic, functional, and quantitative assessment and are relatively complex to perform and interpret. Recently updated Society for Cardiovascular Magnetic Resonance (SCMR) guidelines advocate standardised acquisition and postprocessing to ensure study quality and reproducibility [1, 2]. Manual post-processing is time intensive and can be prone to human error. Therefore, there are active efforts to automate a range of quantitative tasks including ventricular segmentation, myocardial tissue characterisation, and perfusion assessment [3–6]. If validated and made available to the clinical community, automated post-processing pipelines could aid efficiency and consistency of measurements for diagnosis, prognosis, and treatment monitoring.

**Fig. 1:**
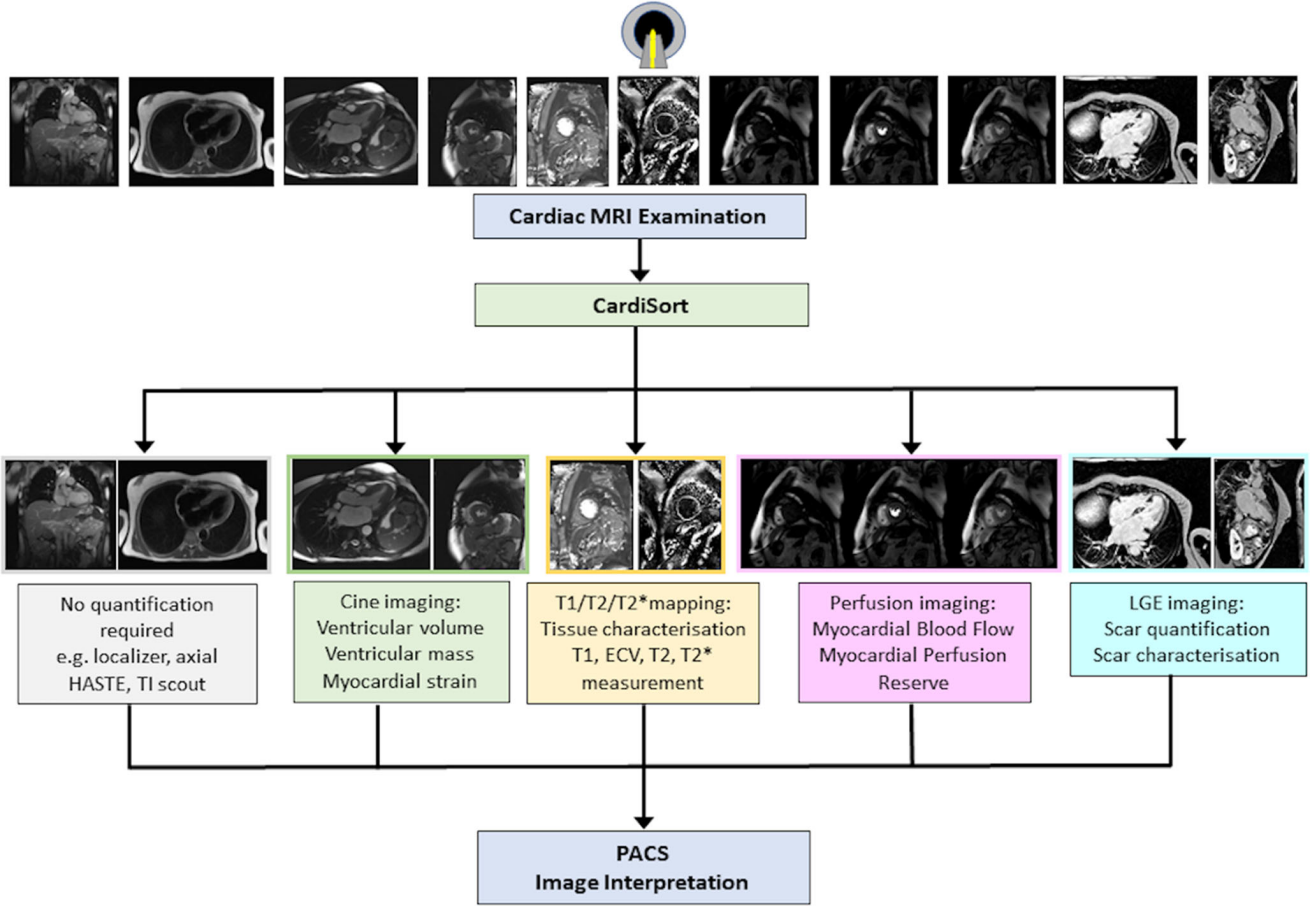
Proposed streamlined clinical workflow for fully automated postprocessing, beginning with an automated cardiac sorting tool (‘CardiSort’). CardiSort would receive images directly from the scanner and classify them, then automatically direct them for further quantitative post-processing as required. Those image types not requiring advanced post-processing would be sent directly to the Picture Archiving and Communication System (PACS). Some examples of multiple automated pipelines that could follow from the initial sorting step include quantification of ventricular volume, function, mass, and myocardial strain from cine imaging; extraction of T1, T2, and T2* values and calculation of extracellular volume (ECV) from T1, T2, and T2* sequences; measurement of myocardial blood flow and myocardial perfusion reserve from stress and rest perfusion imaging, and quantification and characterisation of myocardial scar from late gadolinium-enhanced (LGE) imaging. All images and extracted metrics would then be sent automatically to PACS for image interpretation by a cardiac MR radiologist or cardiologist

Accurate identification of individual cardiac MR sequences is an important first step in directing images to the appropriate post-processing tool. In the clinic, sequence labelling currently depends upon saved scan protocols and/or real-time annotations by the scanning MR technologist, and are thus subject to large variations across centres, making standardisation difficult. Consistent automated sorting would facilitate fully automated post-processing, with a proposed clinical workflow provided in Fig. 1. As well as allowing for prospective automated post-processing, sequence identification can be used to automatically curate large retrospective datasets for training deep learning models. This curation has traditionally relied upon expert manual labour that is time-consuming and costly [7, 8], and an automated means of data curation would facilitate use of larger datasets for more robust tool development and validation.

Van der Voort et al described a convolutional neural network (CNN) approach for automated sorting of 8 brain MRI sequences, achieving greater than 98% accuracy in image labelling [9]. Cardiac MR image sorting is potentially more challenging, due to patient-specific cardiac planes, and variability in sequence design and parameters, and it has not previously been studied in detail. The primary aim of this study was to develop a deep learning tool, CardiSort, to automatically classify a range of clinical cardiac MR sequences using pixel data alone, with real-world cardiac MR data obtained across three different vendors. The model is also made available as an open-source tool for use by the community.

## Materials and methods

### Study design and data

This was a retrospective study to create a model to classify 35 cardiac MRI sequences by sequence type and imaging plane. Anonymised cardiac MRI data was obtained from 4 centres and 3 vendors (Vendor 1, Philips; Vendor 2, Siemens; Vendor 3, General Electric) with institutional ethics approval. A total of 334 randomly sampled studies in 334 patients (224M, 110F, mean ± SD 54.8 ± 15.8 years) were obtained from Centre 1 from 2011 to 2020, with (a) 147 cases scanned at 1.5T (Ingenia, Philips Healthcare); (b) 87 cases scanned at 3T (Achieva, Philips Healthcare); and (c) additional selected sequences obtained from 100 patients on a different 1.5-T vendor system (Aera, Siemens Medical), to supplement training images for vendor 2. Centre 1 common indications for scanning were ischaemic and non-ischaemic cardiomyopathy. Ninety studies in 52 patients with aortic stenosis (31M, 21F, 72.2 ± 7.7 years) were obtained from Centre 2, scanned at 1.5T from 2013 to 2017 (Symphony, Siemens Medical), with studies performed at more than one time point in 37 patients. Forty-eight studies in 48 patients (33M, 15F, 60.0 ± 17.3 years) were obtained from centre 3, scanned at 1.5T from 2017 to 2018 (Optima MR450w, GE Healthcare) with the most common indications of non-ischaemic cardiomyopathy or arrhythmia.

Single vendor training (SVT) was first performed, utilising only data from Centre 1 (which was the largest single vendor training set), followed by multivendor training (MVT), which was retrained from scratch, utilising data from Centres 1 to 3. The final SVT and MVT models were tested on hold-out test data from Centres1 to 3.

**Fig. 2:**
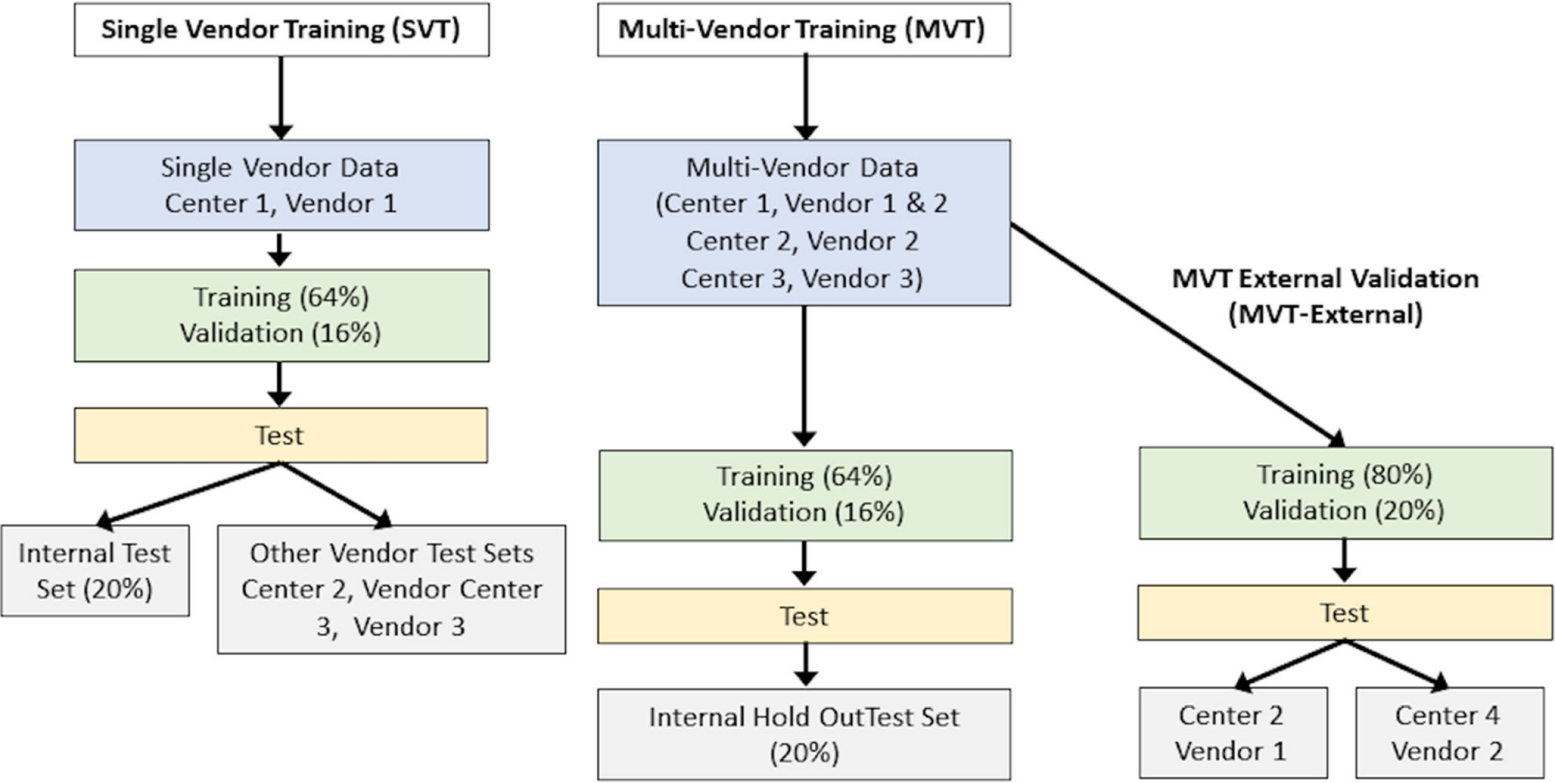
Summary of experiments performed. Initially, a model was developed and trained on data from a single centre (Centre 1) and single vendor (Vendor 1), and tested on data from all 3 vendors. Subsequently, the model was trained on multi-vendor data from Centres 1–3 and tested on a hold-out test set derived from the same multivendor data. Finally, the multivendor trained model was tested on external datasets obtained from systems not used for training

For MVT external validation (MVT_external_), the model was trained on all of the internal data from Centres 1 to 3 as previously described. Testing was performed on 2 external datasets not previously seen by the model: Centre 2 data scanned in 2020 from a different vendor (Vendor 1) 3T system (Achieva, Philips, Healthcare), *n* = 20 patients, 14M, 6F, 58.9 ± 13.5 years; Centre 4 1.5T and 3T Vendor 2 data from 2016 to 2020 (Avanto and Skyra, Siemens Medical), *n* = 60 patients, 31M, 29F, 55.2 ± 14.6 years. The most common clinical indications for external data were non-ischaemic cardiomyopathy, assessment for arrhythmogenic foci, and myocarditis. Experiments performed are summarised in Fig. 2.

#### Pre-processing

All images were obtained in Digital Imaging and Communications in Medicine (DICOM) format and anonymized. Secondary capture images were removed. Slightly different pre-processing using DICOM attributes [10] were required due to vendor-specific differences in the export of sequences with multiple image types, e.g. phase contrast imaging (PC), or multiple planes, e.g. cine imaging. For Vendors 1 and 3, all image types for such sequences were saved combined into a single series; series description, series instance unique identifier (series instance UID), and instance number were used for sorting. For Vendor 2, where multiple image types were saved in separate series but shared a protocol name, protocol name was also included for sorting. Data flow is summarised in Supplementary Figure 1, and sequence labels and final datapoints available are presented by individual magnets in Table 1.

**Table 1:** Sequence and plane of all data, with prevalence of datapoints presented by magnet

		Data used for model training					External Validation		
		Centre 1			Centre 2	Centre 3	Centre 2	Centre 4	Centre 4
Vendor		**Philips**	**Philips**	**Siemens**	**Siemens**	**GE**	**Philips**	**Siemens**	**Siemens**
Field strength (T)		**1.5**	**3**	**1.5**	**1.5**	**1.5**	**1.5**	**1.5**	**3**
Sequence	Plane								
B0 map	Axial	0	36	0	0	0	0	0	0
Cine bSSFP	2-Chamber	275	131	0	181	50	24	13	4
Cine bSSFP	3-Chamber	153	54	0	94	47	27	14	6
Cine bSSFP	4-Chamber	192	145	0	109	50	27	14	15
Cine bSSFP	LVOT	43	3	2	40	0	0	8	5
Cine bSSFP	RVOT	31	4	0	0	7	0	0	10
Cine bSSFP	Short axis	345	194	0	107	48	35	19	13
DBLGE	2-Chamber	127	3	0	0	0	0	0	0
DBLGE	3-Chamber	107	5	0	0	0	0	0	0
DBLGE	4-Chamber	110	4	0	0	0	0	0	0
DBLGE	Short Axis	129	3	0	0	0	0	0	0
EGE	2-Chamber	112	3	0	0	0	0	0	0
EGE	3-Chamber	83	3	0	0	0	0	0	0
EGE	4-Chamber	84	3	0	0	0	0	0	0
FST2	2-Chamber	26	3	25	0	0	3	3	1
FST2	3-Chamber	22	0	27	0	0	0	3	1
FST2	4-Chamber	28	3	25	0	47	2	3	1
FST2	Short axis	30	9	27	0	45	4	0	3
HASTE	Axial	137	11	0	88	47	16	45	14
MOLLI-	Short axis	136	66	94	0	0	0	30	0
MOLLI+	Short axis	141	53	113	0	0	0	29	0
Phase contrast	Aorta	31	7	0	103	11	0	1	12
Phase contrast	MPA	23	5	0	0	8	0	1	9
Perfusion	Short axis	60	83	0	175	0	17	0	0
Scout Imaging	Multiplanar	156	121	0	87	0	8	90	0
T2 mapping bright blood	Short axis	0	0	28	0	0	0	0	0
T2 mapping dark blood	Short axis	26	16	0	0	0	0	0	0
T2* mapping	Short axis	9	33	5	0	0	0	0	0
Test Perfusion (Pre contrast)	Short axis	36	55	0	93	0	15	0	0
TI scout	4-Chamber	30	0	5	0	0	0	0	0
TI scout	Short axis	271	55	172	90	52	18	43	12
WBLGE	2-Chamber	163	60	0	101	47	34	42	5
WBLGE	3-Chamber	141	55	0	98	47	25	33	4
WBLGE	4-Chamber	147	48	0	96	49	30	46	4
WBLGE	Short axis	221	78	0	176	47	21	50	14
Total		**3625**	**1352**	**523**	**1638**	**604**	**306**	**487**	**133**

*bSSFP* balanced steady-state free precession imaging; *LVOT* left ventricular outflow tract, perpendicular to 3-chamber plane; *RVOT* right ventricular outflow tract (oblique sagittal plane); *DBLGE* dark blood late gadolinium-enhanced images (blood pool nulled); *EGE* early gadolinium-enhanced images; *FST2* fat-suppressed T2-weighted imaging; *HASTE* half-Fourier acquisition single-shot turbo spin echo imaging; *MOLLI+* Modified Look Locker Inversion Recovery imaging post contrast; *MOLLI-* native Modified Look Locker Inversion Recovery imaging; *MPA* main pulmonary artery; *TI scout* inversion time scout imaging for late gadolinium-enhanced imaging; *WBLGE* white blood late gadolinium-enhanced images (normal myocardium nulled)

For a unique image series, three images (first, middle, and last as sorted by position and instance number) were selected as input to the model. Three images were chosen as a fixed input size as required by the model. Although this discards images for some image series, it maintains a degree of the temporal, contrast, and spatial information within the series. This was chosen empirically to balance model performance versus model size, based on preliminary experiments. Images were resized to a 256 × 256 pixel array using bilinear interpolation, with array values normalised by the minimum and maximum values to between 0 and 1, per channel. The three images were combined to form a “3-channel” array of shape 256 × 256 × 3 as a single datapoint for model input. For MRI sequences with fewer than 3 images, the first image was repeated once or twice as required.

### Ground truth

Ground truth labels were semi-automatically assigned. Series descriptions for each unique extracted image series were first assigned to classes by a board-certified cardiac radiologist with 15 years’ cardiac MRI experience (SCMR Level 3 equivalent, RPL). All data were then automatically sorted into separate classes based on series description. Of the possible sequence and plane combinations, classes were included in the analysis if at least 20 unique datapoints for that class were present in the entire dataset, resulting in 35 labels incorporating both sequence type and imaging plane (Table 1). The remaining unassigned classes were excluded from further analysis.

Labelled images were then manually reviewed for completeness, label correctness, and diagnostic quality by the same radiologist. Incorrectly labelled images were reassigned to the correct label if present, or excluded if absent. Aberrant congenital anatomy (*n* = 1 patient) and image sets that did not belong to one of the 35 labels, were considered non-diagnostic, or incomplete (2140/26,456 automatically extracted images, 8.1%) were excluded from further analysis. The final datasets underwent repeat review for any misclassification by the same radiologist prior to network training, at least 4 weeks after initial review.

### Data partitioning

In total, 64% of experiment data was chosen for training, 16% for validation, and 20% for testing for SVT and MVT, with MVT_external_ trained on 80% and 20% of the internal data for training and validation respectively, and tested on all of the external data (Supplementary Table 1). Stratified sampling of train, validation, and test sets was performed to eliminate sampling bias, with study-level partitioning to ensure no closely related images from the training set were within validation or test sets for each given class. For MVT, 206 datapoints were excluded from the hold out test set, where data from patients with more than one study for a given class was present in the training data.

### Model

**Fig. 3:**
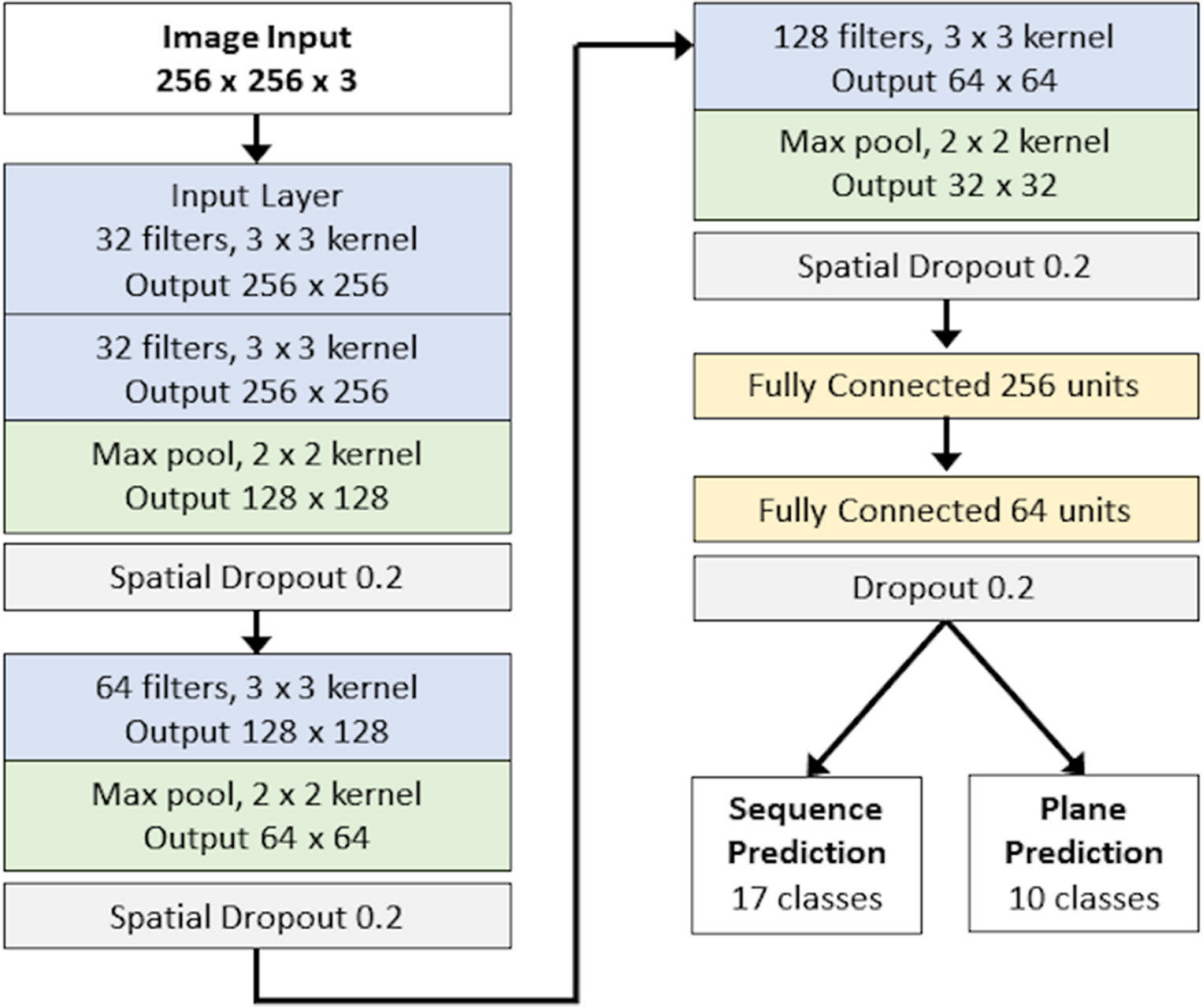
Model architecture. Batch normalization and ReLU activation were used for all hidden layers, with Softmax activation for classification

A 2D CNN, CardiSort, was iteratively developed to evaluate spatial imaging features for two-output (sequence and plane) multiclass classification. All inputs were shuffled prior to presentation to the network. An input layer and three deep convolutional layers with kernel sizes of 3 × 3 were employed for the model with 32, 32, 64, and 128 filters respectively. These were followed by two fully connected layers of 256 and 64 units respectively prior to the output layer, with separate outputs for sequence type and imaging plane (Fig. 3). A ReLU activation function [11] was used for all layers prior to the output layer, with a softmax output used for classification [12]. He-normal weight initialisation was employed [13].

### Training details

Details of training, including data augmentation, selected hyperparameters, and training platform, are provided in Supplementary Methods. Training was performed for 480 epochs, with the lowest summed validation loss observed at least 60 epochs prior to this for all experiments, using categorical cross entropy as the loss metric for both outputs.

The MVT_external_ model is made available at https://github.com/cianmscannell/cardisort, with accompanying code for its application to classifying non-curated data and sorting it into complete imaging series by sequence and plane label.

### Evaluation

Model performance was assessed by overall and per-class classification accuracy (true positive rate) for (a) sequence type, (b) imaging plane, and (c) combined sequence and plane accuracy (Combined). Combined weighted precision, recall, and F1-scores were also calculated. Confusion matrices and gradient-weighted class activation mapping (Grad-CAM) were employed to assess the developed models [14].

## Results

**Fig. 4:**
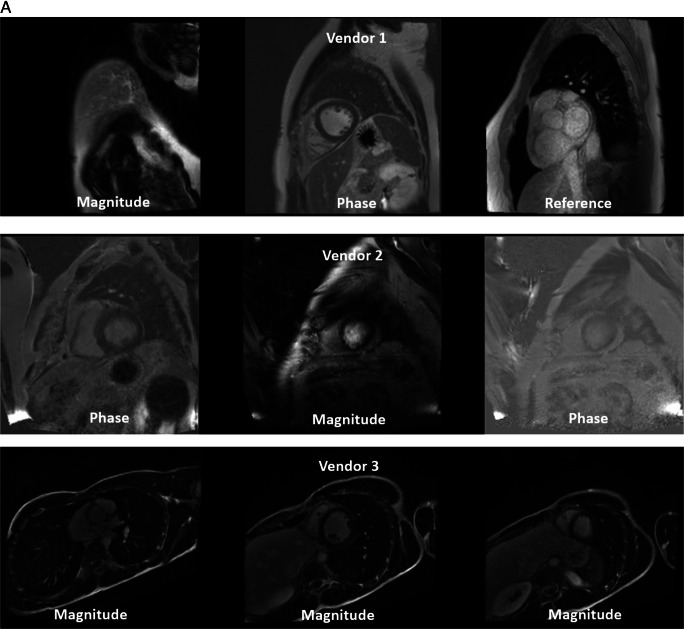

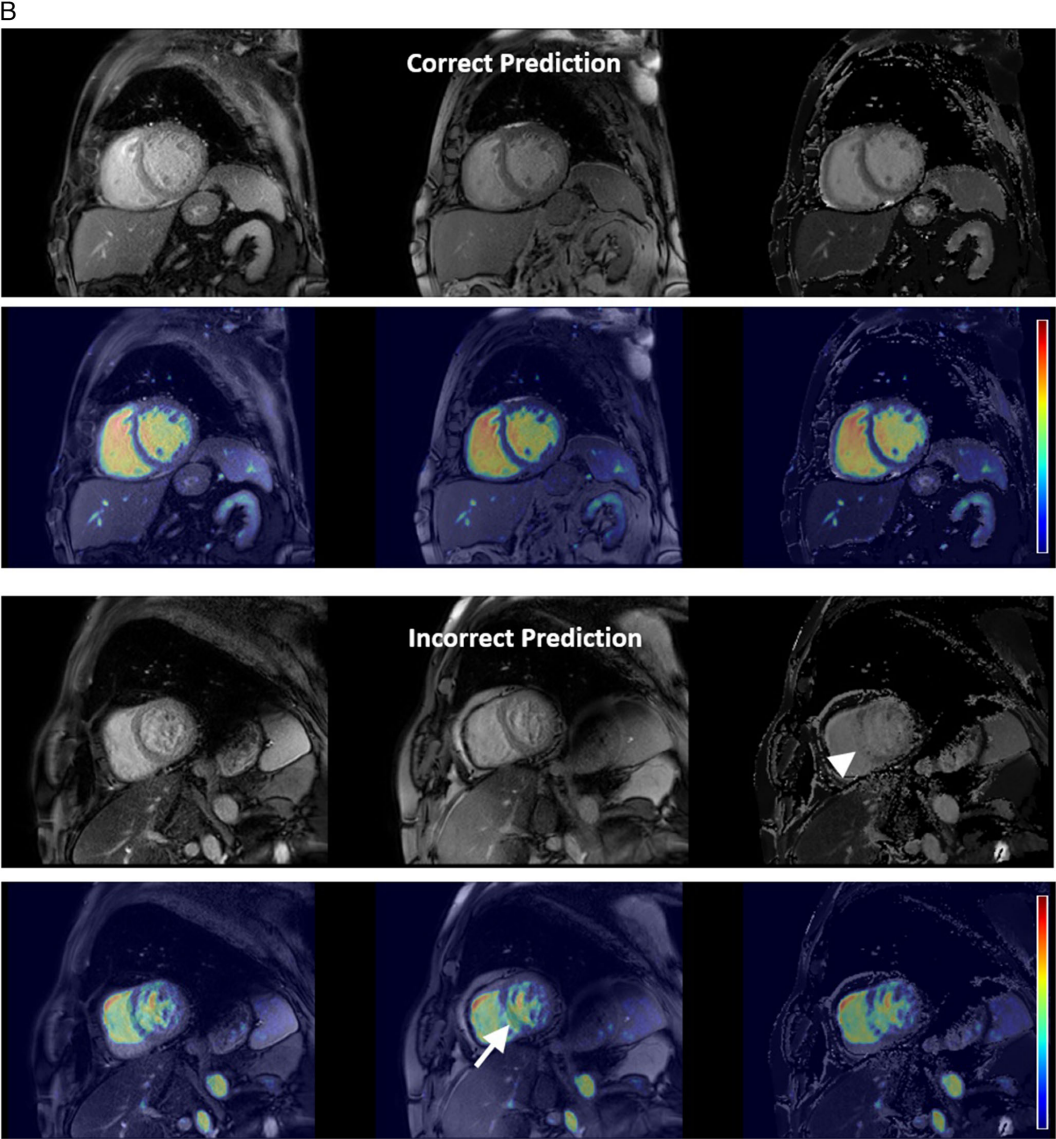

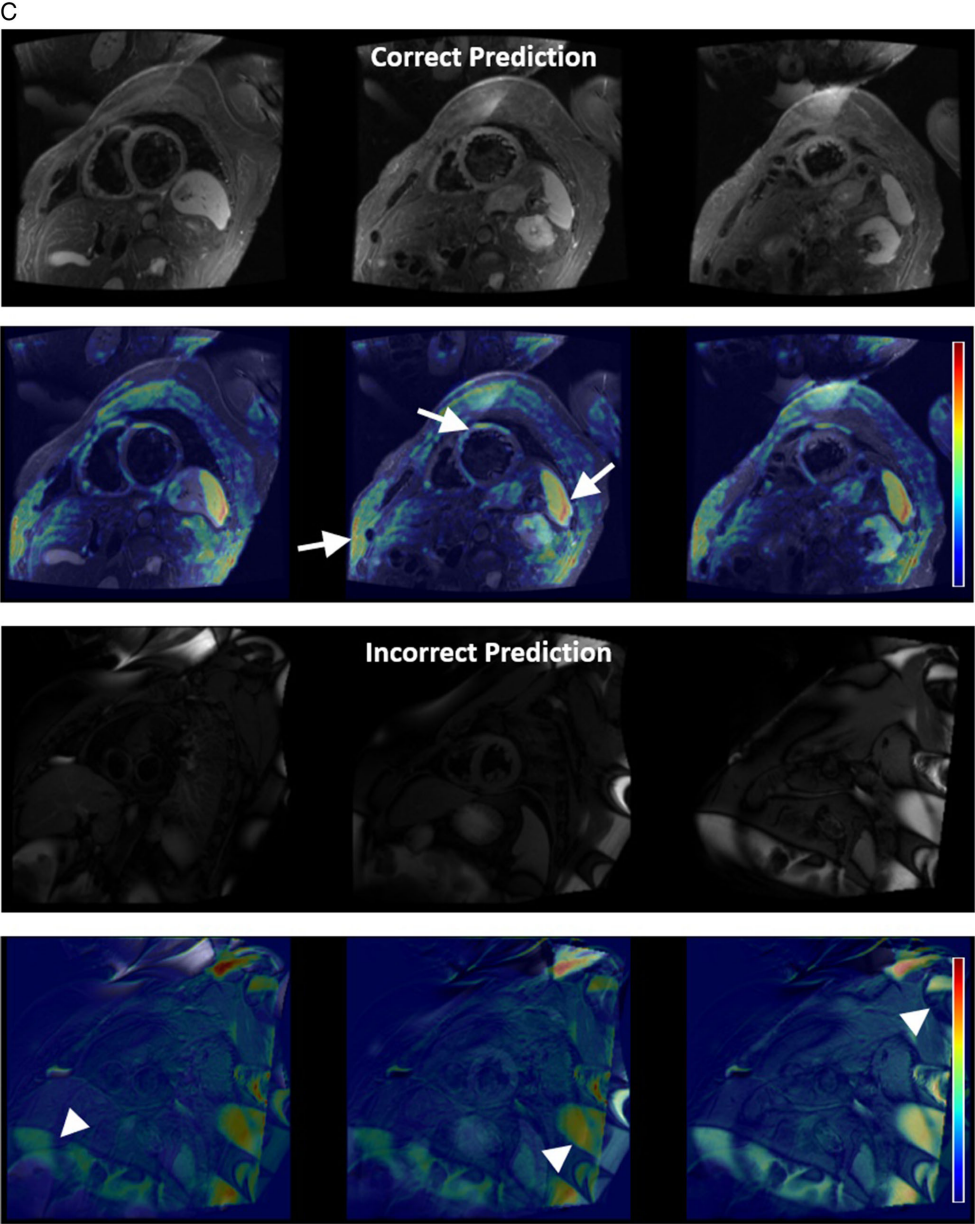

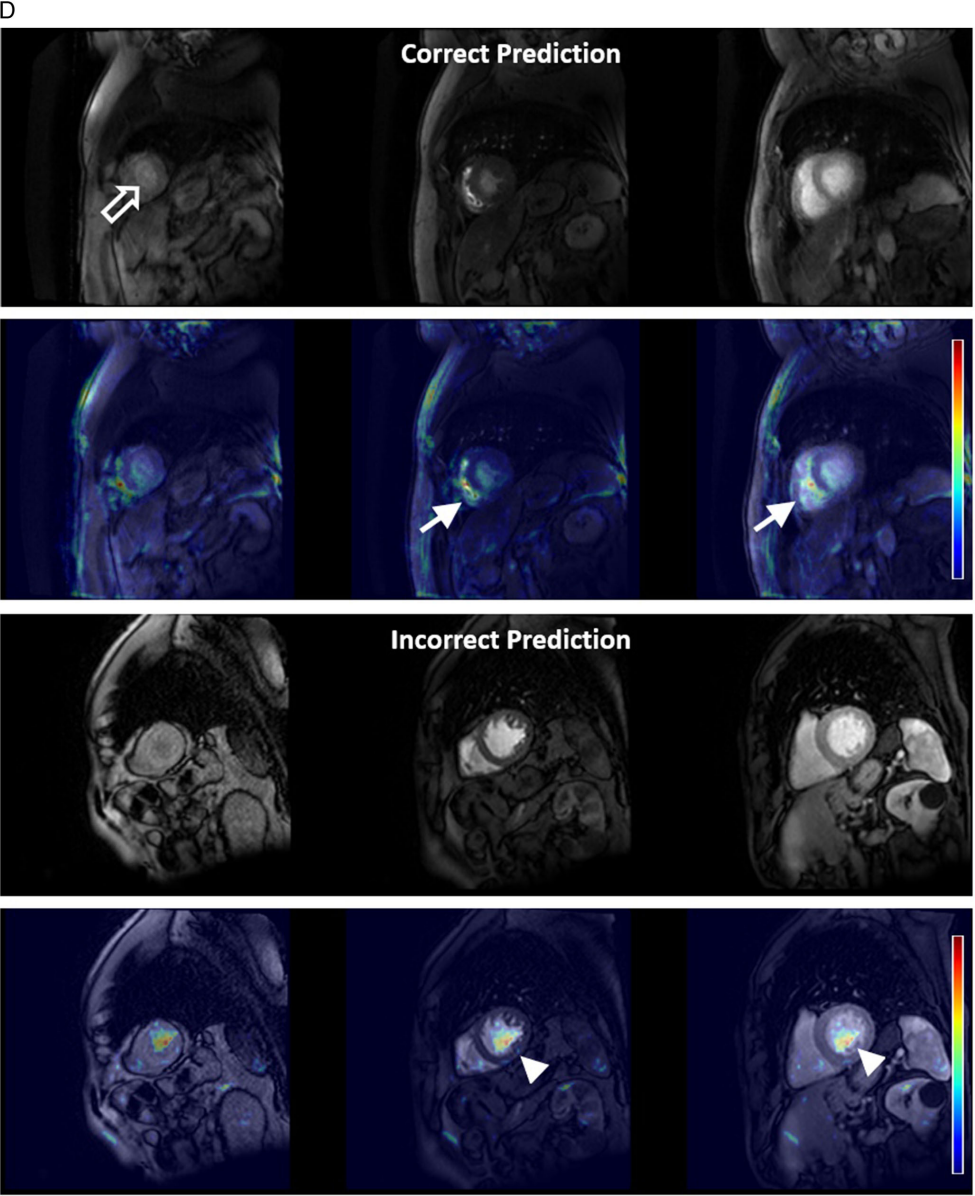
Error analysis. **A** White blood LGE short-axis imaging demonstrating differences between vendors, with 3 image types, magnitude reconstructed inversion recovery (Magnitude), phase reconstructed inversion recovery (Phase), and reference imaging to estimate background phase and surface coil field maps (Reference) images present for Vendor 1. Only the magnitude reconstructed and phase reconstructed images are present for Vendor 2, and only magnitude reconstructed images present for Vendor 3 source data. Also note that differentiation between dark and white blood LGE imaging depends upon appearance of blood and myocardium on magnitude images. **B** Multivendor training native modified Look-Locker Inversion Recovery (MOLLI) T1 mapping, demonstrating a correct and incorrect prediction for Vendor 1 data. In both examples, model attention is focused upon the blood within the ventricles and blood vessels, as indicated by Grad-CAM heat maps superimposed on the original images (second and fourth rows), where red represents a high degree of model attention. However, motion-related artefact is present in the incorrect example, with activation visualized over the inferoseptal segment of the left ventricle (arrows), where myocardial signal appears similar to that of the blood pool on the T1 map (arrowhead), incorrectly predicted as cine short-axis imaging. **C** Multivendor training fat-suppressed T2-weighted short-axis imaging demonstrating a correct and incorrect prediction. For the correct prediction, model attention is focused upon the subcutaneous fat, myocardium, and spleen (arrows), similar to structures a human reader would assess to identify the image type and plane. For the incorrect prediction, the model is focused upon banding artefact related to off-resonance effects at air/soft tissue interfaces (arrowheads). This was predicted as cine short-axis imaging, with cine imaging generally performed with balanced steady-state free precession imaging, which is most prone to banding artefact. **D** Multivendor training external short-axis perfusion imaging examples of a correct prediction from the validation set and incorrect prediction from the external test set. For the correct prediction, model attention is focused upon the right ventricle (arrows) and to a lesser extent the left ventricle, where large relative fluctuations in the contrast of the blood pool of the left and right ventricular chambers are present. For the incorrect prediction, the model is more focused upon the blood pool of the left ventricle (arrowheads), with similar signal within left and right ventricles observed at the mid and basal levels. Note that apical blood pool signal was variable for both training/validation and test dataset, with high signal sometimes observed secondary to flow-related “enhancement” on early perfusion imaging, prior to contrast arrival (hollow arrow)

For SVT, overall test set accuracy, precision, recall, and F1-scores of 81.2%, 0.86, 0.82, and 0.82 were achieved. Test set sequence type and plane accuracies were 85.2% and 93.2% respectively (Table 2). Best performance for Centre 1 data was achieved compared to Centres 2 and 3, where images were acquired on different vendor systems to the training data (per-class results presented in Supplementary Table 2). An example of the differences (in white blood late gadolinium-enhanced images (WBLGE)) between vendors, leading to poor accuracy on unseen vendors for SVT, is shown in Fig. 4a.

**Table 2:** Accuracy, weighted precision, weighted recall and weighted F1-score of the model on test data for each experiment by Centre and Vendor. Combined metrics represents data where both sequence type and plane accuracy are required for a true positive result.

Experiment	Centre	Vendor	Sequence accuracy (%)	Plane accuracy (%)	Combined accuracy (%)	Combined Precision	Combined Recall	Combined F1-score
Single vendor training*	**1**	**Philips**	958/1025 (93.46)	1004/1025 (97.95)	938/1025 (91.51)	0.93	0.92	0.91
	**1 and 2**	**Siemens**	322/438 (73.52)	372/438 (84.93)	295/438 (67.35)	0.79	0.67	0.66
	**3**	**GE**	79/133 (59.40)	112/133 (84.21)	73/133 (54.89)	0.78	0.55	0.53
	**All**	**All**	**1359/1596** **(85.15)**	**1488/1596** **(93.23)**	**1306/1596** **(81.83%)**	**0.86**	**0.82**	**0.82**
Multivendor training**	**1**	**Philips**	976/1025 (95.22)	1003/1025 (97.85)	957/1025 (93.37)	0.94	0.94	0.93
	**1 & 2**	**Siemens**	233/238 (97.90)	236/238 (99.16)	233/238 (97.90)	0.98	0.98	0.98
	**3**	**GE**	132/133 (99.25)	128/133 (96.24)	127/133 (95.49)	0.99	0.95	0.95
	**All**	**All**	**1341/1396** **(96.06)**	**1367/1396** **(97.92)**	**1317/1396** **(94.34)**	**0.95**	**0.94**	**0.94**
Multivendor training – external validation	**2**	**Philips**	262/306 (85.62)	282/306 (92.16)	239/306 (78.10)	0.86	0.78	0.77
	**4**	**Siemens**	596/620 (96.13)	579/620 (93.39)	563/620 (90.81)	0.93	0.91	0.91
	**All external data**		**858/926** **(92.66)**	**861/926** **(92.98)**	**802/926** **(86.61)**	**0.90**	**0.87**	**0.86**

*One class (short-axis T2 bright blood mapping) was omitted from test datasets for single vendor training due to absence of this class in the training data

**Cases where patients overlapped with training data were omitted from the test set for multivendor training

**Fig. 5:**
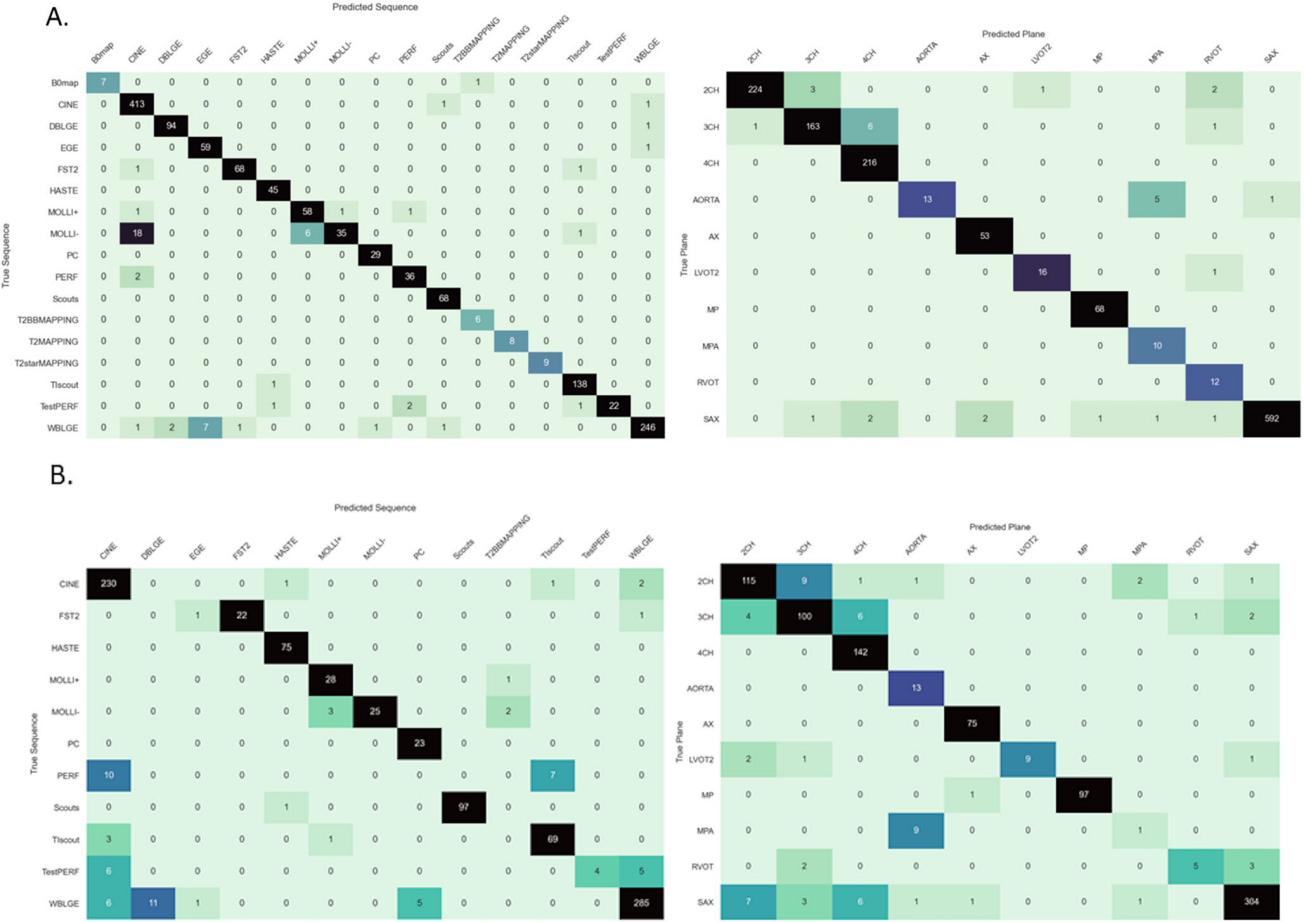
Confusion matrices for multivendor training. **A** Hold-out test set sequence (left) and plane (right) confusion matrices; **B** external data sequence (left) and plane (right) confusion matrices

MVT overall test set accuracy, precision, recall, and F1-scores of 94.3%, 0.95, 0.94, and 0.94 were found. Test set sequence type and plane accuracies were 96.1% and 97.9%, respectively (Table 2). Excellent accuracy was observed for Vendor 2 data (97.9% sequence, 99.2% plane, 97.9% Combined), with near-perfect sequence accuracy for Vendor 3 data (99.3% sequence, 96.2% plane, 95.5% Combined), and stronger plane versus sequence accuracy for Vendor 1 (95.2% sequence, 97.9% plane, 93.4% Combined). The lowest sequence accuracy was observed for Vendor 1 native modified Look Locker inversion recovery (MOLLI) imaging (39.0%), most frequently predicted as cine imaging, compared to 100% sequence accuracy for native MOLLI for Vendor 2, and no Vendor 3 MOLLI data available (Supplementary Table 3). Grad-CAM analysis demonstrated motion-related artefact as a cause of incorrect predictions for this sequence (Fig. 4b). Fat-suppressed T2-weighted (FST2) weighted short axis imaging sequence accuracy was poor for Vendor 2 (4/6 datapoints, 66.7%), with failure of fat suppression and banding artefact observed (Fig. 4c). MVT sequence and plane confusion matrices are presented in Fig. 5a.

MVT_external_ achieved overall test set accuracy, precision, recall, and F1-scores of 86.6%, 0.90, 0.87, and 0.86 on external data (Table 2). Sequence, plane, and Combined accuracies of 85.6%, 92.2%, and 78.1% respectively for Vendor 1, and 96.1%, 93.4%, and 90.8% respectively for Vendor 2 were recorded. High F1-scores for common classes including cine imaging in multiple planes, MOLLI native and post-contrast imaging, TI scout imaging, and WBLGE imaging in all planes were found (Supplementary Table 4).

Excellent overall sequence accuracy of MVT_external_ was observed for cine (94–100%), phase contrast (100%), and long-axis WBLGE (92–100%). Poorest sequence accuracy was observed for (Vendor 1) perfusion imaging (0%), most commonly misclassified as cine (10/17 datapoints) or TI scout sequences (7/17). Differences in sequence parameters, total acquisition time, and contrast protocol (dual-bolus [15] for Vendor 1 training versus single bolus technique for external data), led to visually different image characteristics (Fig. 4d). Relatively low sequence accuracy was observed for WBLGE short axis imaging for Vendor 1 (16/21, 76.2%), classified as dark blood LGE imaging (DBLGE) in 4/21 cases. Suboptimal myocardial nulling and absence of myocardium and blood pool in the included magnitude reconstructed image when positioned distal to the left ventricular apex were observed for incorrect predictions, impacting ability to differentiate between WBLGE and DBLGE (see Fig. 4a, Vendor 1).

MVT_external_ demonstrated high plane prediction accuracy for most common sequence types including cine, MOLLI, TI scout, and WBLGE imaging, strongest for 4 chamber (100%), axial (100%), and short-axis imaging (304/323, 94.1%), and high for 2-chamber (115/129, 89.1%) and 3-chamber (100/113, 88.5%) planes. Poor plane performance was observed for the main pulmonary artery (1/10, 10%), 2-chamber FST2 (4/7, 57.1%), and right ventricular outflow tract (5/10, 50%), with relatively few datapoints available for training. Poor plane performance for short axis FST2 (1/7, 14.3%) was noted, with differences in image export observed between external test (single-slice location per series) and training data (multiple-slice locations per series). Confusion matrices for sequence and plane are presented in Fig. 5b.

## Discussion

In this study, a multivendor deep learning model was developed and validated on external data with high accuracy and F1-scores for identifying commonly performed sequences, particularly cine and WBLGE imaging, and for standard anatomic and cardiac planes. Our work highlights the importance of training the model on a diverse, multi-institutional multivendor dataset, with SVT demonstrating high performance on hold-out test data from the same institution, but poor performance on data from other institutions and vendors, with improved accuracy with MVT on the hold-out multivendor test set.

The variability of clinically used cardiac MRI sequences is also highlighted, impacting model generalizability, exemplified in our study by the decrease in accuracy of MVT from internal test to external data. Though model performance was still very high for common image sequences, it was lower for sequences with differences in parameters and image appearance between external validation and training images, shown by our experience with perfusion imaging. Image artefacts, e.g., misregistration from motion for MOLLI, banding artefact, or failure of fat suppression, also impacted model accuracy. Differences in image export between vendors and institutions also impacted the robustness of the developed model. Vendors provide users with the option to save and export a sequence with multiple slice locations as a single or multiple series. This impacted the model’s ability to predict short-axis FST2 for MVT_external_, where differences in image storage existed between training and external data. Even for sequences that appear relatively uniform, subtle cross-vendor and within-vendor differences may impact model generalizability, as was observed for short axis cine imaging for automated ventricular segmentation [16]. Data augmentation can aid generalizability but does not entirely solve the problem. Greater exposure to a larger variety of data and permutations of image storage for classes with multiple slice locations would likely improve accuracy.

We have made CardiSort available for the scientific community to utilise, facilitating development of fully automated pipelines by allowing automated selection of the requisite image series for processing. We have also provided our trained model weights, should other scientists wish to use transfer learning to include their own data, additional classes, or other magnet systems. Manual selection of the desired sequence is still required in automated cardiac MRI post-processing pipelines described to date [4–6, 17]. Automated extraction from non-curated data would provide a crucial first step to applying AI prospectively in the clinic, rather than retrospectively in a controlled environment. A proposed future clinical workflow would incorporate CardiSort to classify images received directly from the scanner, with sorted images then automatically sent to the appropriate post-processing pipeline for quantitative metrics, e.g. ventricular segmentation [18], myocardial strain [19], perfusion quantification [5], and scar quantification and classification [20], and results of post-processing automatically returned for image interpretation.

There are some limitations to our work. This was a relatively small dataset for a deep learning task, with data augmentation used to expose the network to a greater variety of inputs. Some classes were not available for every vendor in the training data and for external validation. We deliberately chose not to incorporate metadata within model input, which is variable between vendors/centres and may be incomplete or unavailable in publicly available datasets. While the datasets reflect real-world composition of clinical adult cardiac MRI, the model was only trained to recognise standard cardiac anatomy, with insufficient data available for congenital abnormalities.

Our approach, sampling only 3 images per sequence, likely impacted accuracy for predicting sequences that vary temporally, in image contrast or included image type throughout the acquisition, as was observed for short-axis WBLGE for MVT_external_. This represents a practical and efficient means of representing sequences to a model requiring data of a consistent shape for training. Cardiac MRI sequences may comprise a single image or multiple images, and a single or up to three image types, and this approach ensured representation of these. Future work might incorporate other architectures, for example, a 3-dimensional CNN with greater number of input images per sequence and/ or a recurrent neural network to incorporate more sequential information, at the expense of training efficiency.

In conclusion, we have trained a deep learning network on multi-institutional multivendor data to infer 35 unique cardiac MRI sequences by sequence type and imaging plane, with high performance for the most common image types. We have also made our work available for other scientists to use on their own non-curated datasets or to adapt to include additional sequences. Sorting of non-curated data represents a heretofore missing link in the development of efficient and fully automated processing pipelines, essential if they are to be ultimately translated from the research to the clinical domain.

## Supplementary Information

ESM 1 (DOCX 609 kb)

## Abbreviations

2D: Two-dimensional
bSSFP: Balanced steady-state free precession imaging
CNN: Convolutional neural network
DBLGE: Dark blood late gadolinium-enhanced imaging (blood nulled)
DICOM: Digital Imaging and Communications in Medicine
EGE: Early gadolinium-enhanced imaging
FST2: Fat-suppressed T2-weighted imaging
Grad-CAM: Gradient-weighted class activation mapping
HASTE: Half-Fourier acquisition single-shot turbo spin echo imaging
LVOT: Left ventricular outflow tract, perpendicular to 3-chamber plane
MOLLI (+/-): Modified Look Locker inversion recovery imaging (post contrast/native)
MPA: Main pulmonary artery
MVT: Multivendor training
MVT_external_: Multivendor training with external test data
PC: Phase contrast imaging
ReLU: Rectified linear unit
RVOT: Right ventricular outflow tract (oblique sagittal plane)
SCMR: Society for Cardiovascular Magnetic Resonance
SVT: Single vendor training
TI scout: Inversion time scout imaging for late gadolinium-enhanced imaging
UID: Unique identifier
WBLGE: White blood late gadolinium-enhanced imaging (normal myocardium nulled)

## Declarations

### Guarantor

The scientific guarantor of this publication is Ruth P Lim.

### Statistics and biometry

No complex statistical methods were necessary for this paper.

### Methodology

• retrospective

• cross-sectional study

• multicentre study

## References

[1] Kramer CM, Barkhausen J, Bucciarelli-Ducci C, Flamm SD, Kim RJ, Nagel E (2020). Standardized cardiovascular magnetic resonance imaging (CMR) protocols: 2020 update. J Cardiovasc Magn Reson.

[2] Schulz-Menger J, Bluemke DA, Bremerich J (2020). Standardized image interpretation and post-processing in cardiovascular magnetic resonance - 2020 update : Society for Cardiovascular Magnetic Resonance (SCMR): Board of Trustees Task Force on Standardized Post-Processing. J Cardiovasc Magn Reson.

[3] Fahmy AS, El-Rewaidy H, Nezafat M, Nakamori S, Nezafat R (2019). Automated analysis of cardiovascular magnetic resonance myocardial native T1 mapping images using fully convolutional neural networks. J Cardiovasc Magn Reson.

[4] Knott KD, Seraphim A, Augusto JB (2020). The prognostic significance of quantitative myocardial perfusion: an artificial intelligence-based approach using perfusion mapping. Circulation.

[5] Scannell CM, Veta M, Villa ADM (2019). Deep-learning-based preprocessing for quantitative myocardial perfusion MRI. J Magn Reson Imaging.

[6] Tao Q, Yan W, Wang Y (2019). Deep learning-based method for fully automatic quantification of left ventricle function from cine MR images: a multivendor, multicenter study. Radiology.

[7] van Ooijen PMA (2019) Quality and curation of medical images and data. In: Ranschaert ER, Morozov S, Algra PR (eds) Artificial intelligence in medical imaging: opportunities, applications and risks. Springer International Publishing, pp 247–255

[8] Willemink MJ, Koszek WA, Hardell C (2020). Preparing medical imaging data for machine learning. Radiology.

[9] van der Voort SR, Smits M, Klein S (2021). DeepDicomSort: an automatic sorting algorithm for brain magnetic resonance imaging data. Neuroinformatics.

[10] NEMA PS3 / ISO 12052, Digital Imaging and Communications in Medicine (DICOM) Standard, National Electrical Manufacturers Association, Rosslyn, VA, USA. Available via https://www.dicomstandard.org/current. Accessed 03 Aug 2021

[11] Nair V, Hinton G (2010) Rectified Linear Units improve restricted Boltzmann machines. Proceedings of the 27 th International Conference on Machine Learning, Haifa, Israel

[12] Bridle JS (1990) Probabilistic interpretation of feedforward classification network outputs, with relationships to statistical pattern recognition. In: Soulié FF, Hérault J (eds) Neurocomputing. NATO ASI Series (Series F: Computer and Systems Sciences), vol 68. Springer, Berlin, Heidelberg. Available via 10.1007/978-3-642-76153-9_28. Accessed 04 Aug 2021

[13] He K, Zhang X, Ren S, Sun J (2015) Delving deep into rectifiers: surpassing human level performance on ImageNet classification. Proceedings of the IEEE International Conference on Computer Vision (ICCV)

[14] Chollet F (2017) Deep learning for computer vision. In: Deep learning with Python. Manning Publications, pp 119–177

[15] Ishida M, Schuster A, Morton G (2011). Development of a universal dual-bolus injection scheme for the quantitative assessment of myocardial perfusion cardiovascular magnetic resonance. J Cardiovasc Magn Reson.

[16] Campello VM, Gkontra P, Izquierdo C (2021). Multi-centre, multi-vendor and multi-disease cardiac segmentation: The M&Ms Challenge. IEEE Trans Med Imaging.

[17] Böttcher B, Beller E, Busse A (2020). Fully automated quantification of left ventricular volumes and function in cardiac MRI: clinical evaluation of a deep learning-based algorithm. Int J Cardiovasc Imaging.

[18] Scannell CM, Chiribiri A, Veta M (2021) Domain-adversarial learning for multi-centre, multi-vendor, and multi-disease cardiac MR image segmentation. In: Puyol AE, Pop M, Sermesant M et al (eds) Statistical atlases and computational models of the heart. M&Ms and EMIDEC Challenges. STACOM 2020. Springer International Publishing, pp 228–237

[19] Ferdian E, Suinesiaputra A, Fung K (2020). Fully automated myocardial strain estimation from cardiovascular MRI-tagged images using a deep learning framework in the UK Biobank. Radiol Cardiothorac Imaging.

[20] Lourenço A, Kerfoot E, Grigorescu I, Scannell CM, Varela M, Correia TM (2021) Automatic myocardial disease prediction from delayed-enhancement cardiac MRI and clinical information. In: Puyol AE, Pop M, Sermesant M et al (eds) Statistical atlases and computational models of the heart. M&Ms and EMIDEC Challenges. STACOM 2020. Springer International Publishing, pp 334–341

