# Supplementary material for: CardiSort: a convolutional neural network for cross vendor automated sorting of cardiac MR images
Source: Eur Radiol. 2022 Apr 4;32(9):5907–20. doi: 10.1007/s00330-022-08724-4 (PMC9381634; doi:10.1007/s00330-022-08724-4)
Supplement: Supplementary file 1 — (DOCX 609 kb) [file 330_2022_8724_MOESM1_ESM.docx]

**Supplementary Material**

**Supplementary Table 1.** Number of patients by partition for single vendor training (SVT), multivendor training (MVT) and multivendor training with external test data (MVT_external_). SVT and MVT test data included test set data from Centres 2 and 3 as well as hold out test subjects from Centre 1. MVT_external_ test data comprised external test data alone.

|  | **SVT** | | | **MVT** | | | **MVT_external_** | | |
| --- | --- | --- | --- | --- | --- | --- | --- | --- | --- |
|  | **Training** | **Validation** | **Test** | **Training** | **Validation** | **Test*** | **Training** | **Validation** | **Test** |
| **B0map_AX** | 20 | 5 | 7 | 20 | 5 | 7 | 25 | 7 |  |
| **CINE_2CH** | 140 | 36 | 71 | 209 | 57 | 63 | 262 | 72 | 34 |
| **CINE_3CH** | 122 | 31 | 65 | 191 | 51 | 55 | 239 | 65 | 32 |
| **CINE_4CH** | 145 | 37 | 74 | 216 | 58 | 60 | 272 | 74 | 44 |
| **CINE_LVOT** | 26 | 8 | 18 | 49 | 15 | 17 | 63 | 18 | 12 |
| **CINE_RVOT** | 18 | 5 | 9 | 22 | 6 | 9 | 28 | 9 | 10 |
| **CINE_SAX** | 147 | 38 | 74 | 221 | 60 | 65 | 272 | 74 | 45 |
| **DBLGE_2CH** | 68 | 18 | 22 | 68 | 18 | 22 | 86 | 22 |  |
| **DBLGE_3CH** | 61 | 17 | 21 | 61 | 17 | 21 | 78 | 21 |  |
| **DBLGE_4CH** | 65 | 18 | 22 | 65 | 18 | 22 | 83 | 22 |  |
| **DBLGE_SAX** | 67 | 18 | 22 | 67 | 18 | 22 | 85 | 22 |  |
| **EGE_2CH** | 66 | 18 | 22 | 66 | 18 | 22 | 84 | 22 |  |
| **EGE_3CH** | 52 | 14 | 18 | 52 | 14 | 18 | 66 | 18 |  |
| **EGE_4CH** | 53 | 15 | 18 | 53 | 15 | 18 | 68 | 18 |  |
| **FST2_2CH** | 16 | 5 | 12 | 32 | 9 | 12 | 41 | 12 | 7 |
| **FST2_3CH** | 12 | 5 | 11 | 28 | 9 | 11 | 37 | 11 | 4 |
| **FST2_4CH** | 17 | 5 | 20 | 61 | 17 | 20 | 78 | 20 | 6 |
| **FST2_SAX** | 20 | 5 | 22 | 64 | 17 | 22 | 81 | 22 | 3 |
| **HASTE_AX** | 89 | 24 | 57 | 161 | 46 | 44 | 200 | 57 | 75 |
| **MOLLI+_SAX** | 94 | 24 | 50 | 153 | 39 | 50 | 192 | 50 | 28 |
| **MOLLI-_SAX** | 110 | 28 | 54 | 170 | 43 | 54 | 213 | 54 | 30 |
| **PC_AORTA** | 22 | 6 | 27 | 69 | 21 | 15 | 83 | 27 | 13 |
| **PC_MPA** | 14 | 5 | 8 | 17 | 6 | 8 | 23 | 8 | 9 |
| **PERF_SAX** | 46 | 12 | 32 | 92 | 25 | 20 | 110 | 32 | 12 |
| **Scouts_MP** | 147 | 38 | 64 | 188 | 51 | 57 | 233 | 64 | 52 |
| **T2BBMAPPING_SAX** |  |  |  | 17 | 5 | 6 | 22 | 6 |  |
| **T2MAPPING_SAX** | 22 | 7 | 8 | 22 | 7 | 8 | 29 | 8 |  |
| **T2starMAPPING_SAX** | 22 | 6 | 8 | 25 | 7 | 8 | 32 | 8 |  |
| **TestPERF_SAX** | 43 | 12 | 32 | 86 | 25 | 20 | 104 | 32 | 10 |
| **TIscout_4CH** | 14 | 4 | 7 | 16 | 5 | 7 | 21 | 7 |  |
| **TIscout_SAX** | 113 | 30 | 84 | 248 | 67 | 72 | 309 | 84 | 68 |
| **WBLGE_2CH** | 109 | 29 | 63 | 180 | 51 | 51 | 226 | 63 | 63 |
| **WBLGE_3CH** | 103 | 26 | 60 | 172 | 49 | 52 | 213 | 68 | 54 |
| **WBLGE_4CH** | 104 | 28 | 61 | 176 | 49 | 51 | 217 | 61 | 65 |
| **WBLGE_SAX** | 108 | 29 | 62 | 179 | 51 | 55 | 223 | 62 | 74 |

***** MVT test data excluded data from patients for any given class already represented within the training data by a study from a different time point (Centre 2, Vendor 2, n=37 patients).

**Supplementary Table 2.** Single Vendor Training per class sequence, plane and overall accuracy by Vendor, and overall weighted precision, recall and F1-scores on the hold out test set.

|  | **Sequence Accuracy (%)** | | | | **Plane Accuracy (%)** | | | | **Combined Accuracy (%)** | | | | **Combined**  **Precision** | **Combined**  **Recall** | **Combined**  **F1-score** |
| --- | --- | --- | --- | --- | --- | --- | --- | --- | --- | --- | --- | --- | --- | --- | --- |
| **Vendor:** | **1** | **2** | **3** |  | **1** | **2** | **3** |  | **1** | **2** | **3** |  |  |  |  |
| **Label (below)** | **Philips** | **Siemens** | **GE** | **All** | **Philips** | **Siemens** | **GE** | **All** | **Philips** | **Siemens** | **GE** | **All** |  |  |  |
| **B0map_AX** | 100.00 |  |  | 100.00 | 100.00 |  |  | 100.00 | 100.00 |  |  | 100.00 | 0.89 | 1.00 | 0.94 |
| **CINE_2CH** | 100.00 | 100.00 | 100.00 | 100.00 | 90.36 | 97.14 | 90.00 | 92.19 | 90.36 | 97.14 | 90.00 | 92.19 | 0.96 | 0.92 | 0.94 |
| **CINE_3CH** | 100.00 | 100.00 | 100.00 | 100.00 | 97.44 | 76.19 | 80.00 | 88.57 | 97.44 | 76.19 | 80.00 | 88.57 | 0.97 | 0.92 | 0.94 |
| **CINE_4CH** | 100.00 | 100.00 | 100.00 | 100.00 | 100.00 | 95.65 | 90.91 | 98.15 | 100.00 | 95.65 | 90.91 | 98.15 | 0.93 | 0.98 | 0.95 |
| **CINE_LVOT** | 100.00 | 100.00 |  | 100.00 | 88.89 | 44.44 |  | 66.67 | 88.89 | 44.44 |  | 66.67 | 1.00 | 0.67 | 0.80 |
| **CINE_RVOT** | 100.00 |  | 100.00 | 100.00 | 100.00 |  | 100.00 | 100.00 | 100.00 |  | 100.00 | 100.00 | 0.48 | 1.00 | 0.65 |
| **CINE_SAX** | 98.17 | 80.95 | 100.00 | 95.71 | 99.08 | 80.95 | 100.00 | 96.43 | 97.25 | 76.19 | 100.00 | 94.29 | 0.66 | 0.94 | 0.77 |
| **DBLGE_2CH** | 100.00 |  |  | 100.00 | 100.00 |  |  | 100.00 | 100.00 |  |  | 100.00 | 1.00 | 1.00 | 1.00 |
| **DBLGE_3CH** | 95.83 |  |  | 95.83 | 100.00 |  |  | 100.00 | 95.83 |  |  | 95.83 | 0.96 | 0.96 | 0.96 |
| **DBLGE_4CH** | 95.65 |  |  | 95.65 | 100.00 |  |  | 100.00 | 95.65 |  |  | 95.65 | 0.85 | 0.96 | 0.90 |
| **DBLGE_SAX** | 95.83 |  |  | 95.83 | 100.00 |  |  | 100.00 | 95.83 |  |  | 95.83 | 0.85 | 0.96 | 0.90 |
| **EGE_2CH** | 95.83 |  |  | 95.83 | 100.00 |  |  | 100.00 | 95.83 |  |  | 95.83 | 0.66 | 0.96 | 0.78 |
| **EGE_3CH** | 100.00 |  |  | 100.00 | 100.00 |  |  | 100.00 | 100.00 |  |  | 100.00 | 0.78 | 1.00 | 0.88 |
| **EGE_4CH** | 94.44 |  |  | 94.44 | 100.00 |  |  | 100.00 | 94.44 |  |  | 94.44 | 0.74 | 0.94 | 0.83 |
| **FST2_2CH** | 100.00 | 40.00 | 100.00 | 76.92 | 100.00 | 60.00 | 100.00 | 84.62 | 100.00 | 20.00 | 100.00 | 69.23 | 0.69 | 0.69 | 0.69 |
| **FST2_3CH** | 100.00 | 100.00 | 100.00 | 100.00 | 50.00 | 100.00 | 0.00 | 66.67 | 50.00 | 100.00 | 0.00 | 66.67 | 0.38 | 0.67 | 0.48 |
| **FST2_4CH** | 100.00 | 100.00 | 100.00 | 100.00 | 83.33 | 80.00 | 100.00 | 90.00 | 83.33 | 80.00 | 100.00 | 90.00 | 0.69 | 0.90 | 0.78 |
| **FST2_SAX** | 90.00 | 50.00 | 66.67 | 72.00 | 100.00 | 83.33 | 100.00 | 96.00 | 90.00 | 50.00 | 66.67 | 72.00 | 0.75 | 0.72 | 0.73 |
| **HASTE_AX** | 100.00 | 100.00 | 60.00 | 93.10 | 100.00 | 100.00 | 60.00 | 93.10 | 100.00 | 100.00 | 60.00 | 93.10 | 0.87 | 0.93 | 0.90 |
| **MOLLI+_SAX** | 88.89 | 0.00 |  | 52.46 | 100.00 | 100.00 |  | 100.00 | 88.89 | 0.00 |  | 52.46 | 0.82 | 0.52 | 0.64 |
| **MOLLI-_SAX** | 34.15 | 0.00 |  | 23.33 | 97.56 | 89.47 |  | 95.00 | 34.15 | 0.00 |  | 23.33 | 1.00 | 0.23 | 0.38 |
| **PC_AORTA** | 100.00 | 5.26 | 0.00 | 30.30 | 88.89 | 5.26 | 0.00 | 27.27 | 88.89 | 5.26 | 0.00 | 27.27 | 0.75 | 0.27 | 0.40 |
| **PC_MPA** | 100.00 |  | 0.00 | 80.00 | 87.50 |  | 0.00 | 70.00 | 87.50 |  | 0.00 | 70.00 | 0.54 | 0.70 | 0.61 |
| **PERF_SAX** | 89.66 | 88.57 |  | 89.06 | 100.00 | 97.14 |  | 98.44 | 89.66 | 88.57 |  | 89.06 | 0.89 | 0.89 | 0.89 |
| **Scouts_MP** | 96.61 | 94.44 |  | 96.10 | 100.00 | 94.44 |  | 98.70 | 96.61 | 94.44 |  | 96.10 | 0.91 | 0.96 | 0.94 |
| **T2MAPPING_SAX** | 100.00 |  |  | 100.00 | 100.00 |  |  | 100.00 | 100.00 |  |  | 100.00 | 0.73 | 1.00 | 0.84 |
| **T2starMAPPING_SAX** | 62.50 | 0.00 |  | 55.56 | 100.00 | 100.00 |  | 100.00 | 62.50 | 0.00 |  | 55.56 | 1.00 | 0.56 | 0.71 |
| **TestPERF_SAX** | 95.24 | 73.68 |  | 85.00 | 100.00 | 100.00 |  | 100.00 | 95.24 | 73.68 |  | 85.00 | 0.45 | 0.85 | 0.59 |
| **TIscout_4CH** | 100.00 | 100.00 |  | 100.00 | 100.00 | 100.00 |  | 100.00 | 100.00 | 100.00 |  | 100.00 | 0.83 | 1.00 | 0.91 |
| **TIscout_SAX** | 100.00 | 63.16 | 84.62 | 83.80 | 98.61 | 91.23 | 100.00 | 95.77 | 98.61 | 61.40 | 84.62 | 82.39 | 0.97 | 0.82 | 0.89 |
| **WBLGE_2CH** | 88.64 | 100.00 | 0.00 | 80.26 | 97.73 | 81.82 | 80.00 | 90.79 | 86.36 | 81.82 | 0.00 | 73.68 | 0.90 | 0.74 | 0.81 |
| **WBLGE_3CH** | 92.31 | 95.24 | 10.00 | 81.43 | 97.44 | 85.71 | 100.00 | 94.29 | 89.74 | 80.95 | 10.00 | 75.71 | 0.93 | 0.76 | 0.83 |
| **WBLGE_4CH** | 91.89 | 85.71 | 10.00 | 77.94 | 100.00 | 85.71 | 70.00 | 91.18 | 91.89 | 85.71 | 0.00 | 76.47 | 0.93 | 0.76 | 0.84 |
| **WBLGE_SAX** | 86.44 | 75.00 | 0.00 | 74.26 | 100.00 | 71.88 | 100.00 | 91.09 | 86.44 | 62.50 | 0.00 | 70.30 | 0.85 | 0.70 |  |

bSSFP = balanced steady state free precession imaging

CH = chamber

LVOT = left ventricular outflow tract, perpendicular to 3-chamber plane

RVOT = right ventricular outflow tract (oblique sagittal plane)

DBLGE = dark blood late gadolinium enhanced images, with nulling of blood pool

EGE = early gadolinium enhanced images

FST2 = fat suppressed T2 weighted imaging

HASTE = half Fourier acquisition single shot turbo spin echo imaging

MOLLI+ = Modified Look Locker Inversion Recovery imaging post contrast

MOLLI- = native Modified Look Locker Inversion Recovery imaging

MPA = main pulmonary artery

PERF = perfusion imaging

TestPERF = test perfusion imaging (no contrast administered)

SAX = short axis

TI scout = inversion time scout imaging for late gadolinium enhanced imaging

WBLGE = white blood late gadolinium enhanced images, with nulling of normal myocardium

**Supplementary Table 3.** Multi-Vendor Training per class sequence, plane and overall accuracy by Vendor, and overall weighted precision, recall and F1-scores on the hold out test set

|  | **Sequence Accuracy (%)** | | | | **Plane Accuracy (%)** | | | | **Combined Accuracy (%)** | | | | **Combined**  **Precision** | **Combined**  **Recall** | **Combined**  **F1-score** |
| --- | --- | --- | --- | --- | --- | --- | --- | --- | --- | --- | --- | --- | --- | --- | --- |
| **Vendor:** | **1** | **2** | **3** |  | **1** | **2** | **3** |  | **1** | **2** | **3** |  |  |  |  |
| **Label (below)** | **Philips** | **Siemens** | **GE** | **All** | **Philips** | **Siemens** | **GE** | **All** | **Philips** | **Siemens** | **GE** | **All** |  |  |  |
| **B0map_AX** | 87.50 |  |  | 87.50 | 100.00 |  |  | 100.00 | 87.50 |  |  | 87.50 | 1.00 | 0.88 | 0.93 |
| **CINE_2CH** | 100.00 | 100.00 | 100.00 | 100.00 | 96.39 | 100.00 | 100.00 | 97.66 | 96.39 | 100.00 | 100.00 | 97.66 | 1.00 | 0.97 | 0.99 |
| **CINE_3CH** | 100.00 | 100.00 | 100.00 | 100.00 | 92.31 | 100.00 | 100.00 | 95.71 | 92.31 | 100.00 | 100.00 | 95.71 | 1.00 | 0.95 | 0.97 |
| **CINE_4CH** | 100.00 | 100.00 | 100.00 | 100.00 | 100.00 | 100.00 | 100.00 | 100.00 | 100.00 | 100.00 | 100.00 | 100.00 | 0.98 | 1.00 | 0.99 |
| **CINE_LVOT** | 100.00 | 100.00 |  | 100.00 | 88.89 | 100.00 |  | 94.44 | 88.89 | 100.00 |  | 94.44 | 0.94 | 0.94 | 0.94 |
| **CINE_RVOT** | 100.00 |  | 100.00 | 100.00 | 100.00 |  | 100.00 | 100.00 | 100.00 |  | 100.00 | 100.00 | 0.71 | 1.00 | 0.83 |
| **CINE_SAX** | 99.08 | 100.00 | 90.00 | 98.57 | 99.08 | 100.00 | 100.00 | 99.29 | 98.17 | 100.00 | 90.00 | 97.86 | 0.85 | 0.98 | 0.91 |
| **DBLGE_2CH** | 100.00 |  |  | 100.00 | 100.00 |  |  | 100.00 | 100.00 |  |  | 100.00 | 0.96 | 1.00 | 0.98 |
| **DBLGE_3CH** | 100.00 |  |  | 100.00 | 95.83 |  |  | 95.83 | 95.83 |  |  | 95.83 | 1.00 | 0.96 | 0.98 |
| **DBLGE_4CH** | 100.00 |  |  | 100.00 | 100.00 |  |  | 100.00 | 100.00 |  |  | 100.00 | 0.96 | 1.00 | 0.98 |
| **DBLGE_SAX** | 95.83 |  |  | 95.83 | 100.00 |  |  | 100.00 | 95.83 |  |  | 95.83 | 0.96 | 0.96 | 0.96 |
| **EGE_2CH** | 100.00 |  |  | 100.00 | 100.00 |  |  | 100.00 | 100.00 |  |  | 100.00 | 0.83 | 1.00 | 0.91 |
| **EGE_3CH** | 100.00 |  |  | 100.00 | 94.44 |  |  | 94.44 | 94.44 |  |  | 94.44 | 0.94 | 0.94 | 0.94 |
| **EGE_4CH** | 94.44 |  |  | 94.44 | 100.00 |  |  | 100.00 | 94.44 |  |  | 94.44 | 0.89 | 0.94 | 0.92 |
| **FST2_2CH** | 100.00 | 100.00 | 100.00 | 100.00 | 100.00 | 100.00 | 0.00 | 92.31 | 100.00 | 100.00 | 0.00 | 92.31 | 1.00 | 0.92 | 0.96 |
| **FST2_3CH** | 100.00 | 100.00 | 100.00 | 100.00 | 83.33 | 100.00 | 100.00 | 91.67 | 83.33 | 100.00 | 100.00 | 91.67 | 0.92 | 0.92 | 0.92 |
| **FST2_4CH** | 100.00 | 100.00 | 100.00 | 100.00 | 100.00 | 100.00 | 100.00 | 100.00 | 100.00 | 100.00 | 100.00 | 100.00 | 0.91 | 1.00 | 0.95 |
| **FST2_SAX** | 100.00 | 66.67 | 100.00 | 92.00 | 100.00 | 100.00 | 100.00 | 100.00 | 100.00 | 66.67 | 100.00 | 92.00 | 1.00 | 0.92 | 0.96 |
| **HASTE_AX** | 100.00 | 100.00 | 100.00 | 100.00 | 100.00 | 100.00 | 100.00 | 100.00 | 100.00 | 100.00 | 100.00 | 100.00 | 0.96 | 1.00 | 0.98 |
| **MOLLI+_SAX** | 94.44 | 96.00 |  | 95.08 | 100.00 | 100.00 |  | 100.00 | 94.44 | 96.00 |  | 95.08 | 0.91 | 0.95 | 0.93 |
| **MOLLI-_SAX** | 39.02 | 100.00 |  | 58.33 | 97.56 | 100.00 |  | 98.33 | 39.02 | 100.00 |  | 58.33 | 0.97 | 0.58 | 0.73 |
| **PC_AORTA** | 100.00 | 100.00 | 100.00 | 100.00 | 77.78 | 100.00 | 20.00 | 81.82 | 77.78 | 100.00 | 20.00 | 81.82 | 1.00 | 0.68 | 0.81 |
| **PC_MPA** | 100.00 |  | 100.00 | 100.00 | 100.00 |  | 100.00 | 100.00 | 100.00 |  | 100.00 | 100.00 | 0.62 | 1.00 | 0.77 |
| **PERF_SAX** | 93.10 | 97.14 |  | 95.31 | 100.00 | 100.00 |  | 100.00 | 93.10 | 97.14 |  | 95.31 | 0.92 | 0.95 | 0.94 |
| **Scouts_MP** | 100.00 | 100.00 |  | 100.00 | 100.00 | 100.00 |  | 100.00 | 100.00 | 100.00 |  | 100.00 | 0.99 | 1.00 | 0.99 |
| **T2BBMAPPING_SAX** |  | 100.00 |  | 100.00 |  | 100.00 |  | 100.00 |  | 100.00 |  | 100.00 | 1.00 | 1.00 | 1.00 |
| **T2MAPPING_SAX** | 100.00 |  |  | 100.00 | 100.00 |  |  | 100.00 | 100.00 |  |  | 100.00 | 1.00 | 1.00 | 1.00 |
| **T2starMAPPING_SAX** | 100.00 | 100.00 |  | 100.00 | 100.00 | 100.00 |  | 100.00 | 100.00 | 100.00 |  | 100.00 | 1.00 | 1.00 | 1.00 |
| **TestPERF_SAX** | 80.95 | 100.00 |  | 90.00 | 95.24 | 94.74 |  | 95.00 | 80.95 | 94.74 |  | 87.50 | 1.00 | 0.85 | 0.92 |
| **TIscout_4CH** | 100.00 | 100.00 |  | 100.00 | 100.00 | 100.00 |  | 100.00 | 100.00 | 100.00 |  | 100.00 | 0.83 | 1.00 | 0.91 |
| **TIscout_SAX** | 100.00 | 98.25 |  | 99.30 | 97.22 | 98.25 | 100.00 | 97.89 | 97.22 | 98.25 | 100.00 | 97.89 | 0.98 | 0.98 | 0.98 |
| **WBLGE_2CH** | 86.36 | 100.00 |  | 92.11 | 95.45 | 100.00 | 100.00 | 97.37 | 81.82 | 100.00 | 100.00 | 89.47 | 0.98 | 0.87 | 0.92 |
| **WBLGE_3CH** | 97.44 | 100.00 |  | 98.57 | 94.87 | 100.00 | 100.00 | 97.14 | 92.31 | 100.00 | 100.00 | 95.71 | 0.97 | 0.95 | 0.96 |
| **WBLGE_4CH** | 94.59 | 100.00 |  | 97.06 | 100.00 | 100.00 | 100.00 | 100.00 | 94.59 | 100.00 | 100.00 | 97.06 | 0.96 | 0.96 | 0.96 |
| **WBLGE_SAX** | 94.92 | 96.88 |  | 96.04 | 98.31 | 96.88 | 100.00 | 98.02 | 94.92 | 96.88 | 100.00 | 96.04 | 0.98 | 0.95 | 0.96 |

bSSFP = balanced steady state free precession imaging

CH = chamber

LVOT = left ventricular outflow tract, perpendicular to 3-chamber plane

RVOT = right ventricular outflow tract (oblique sagittal plane)

DBLGE = dark blood late gadolinium enhanced images, with nulling of blood pool

EGE = early gadolinium enhanced images

FST2 = fat suppressed T2 weighted imaging

HASTE = half Fourier acquisition single shot turbo spin echo imaging

MOLLI+ = Modified Look Locker Inversion Recovery imaging post contrast

MOLLI- = native Modified Look Locker Inversion Recovery imaging

MPA = main pulmonary artery

PERF = perfusion imaging

SAX = short axis

T2BBMAPPING = T2 bright blood mapping

TestPERF = test perfusion imaging (no contrast administered)

TI scout = inversion time scout imaging for late gadolinium enhanced imaging

WBLGE = white blood late gadolinium enhanced images, with nulling of normal myocardium

**Supplementary Table 4.** Multi-Vendor Training per class sequence, plane and overall accuracy by Vendor, and overall weighted precision, recall and F1-scores on external validation data by Vendor. Fraction and percentage of correct datapoints per class and vendor are provided.

|  | **Sequence Accuracy (%)** | | | **Plane Accuracy (%)** | | | **Combined Accuracy (%)** | | | **Combined**  **Precision** | **Combined**  **Recall** | **Combined**  **F1-score** |
| --- | --- | --- | --- | --- | --- | --- | --- | --- | --- | --- | --- | --- |
| **Vendor:** | **1** | **2** | **1 & 2** | **1** | **2** | **1 & 2** | **1** | **2** | **1 & 2** |  |  |  |
| **Label (below)** | **Philips** | **Siemens** | **Both** | **Philips** | **Siemens** | **Both** | **Philips** | **Siemens** | **Both** |  |  |  |
| **CINE_2CH** | 24/24  (100.00) | 17/17  (100.00) | 41/41  (100.00) | 22/24  (91.67) | 17/17  (100.00) | 39/41  (95.12) | 22/24  (91.67) | 17/17  (100.00) | 39/41  (95.12) | 0.87 | 0.95 | 0.91 |
| **CINE_3CH** | 27/27  (100.00) | 20/20  (100.00) | 47/47  (100.00) | 21/27  (77.78) | 20/20  (100.00) | 41/47  (87.23) | 21/27  (77.78) | 20/20  (100.00) | 41/47  (87.23) | 0.91 | 0.87 | 0.89 |
| **CINE_4CH** | 27/27  (100.00) | 29/29  (100.00) | 56/56  (100.00) | 27/27  (100.00) | 29/29  (100.00) | 56/56  (100.00) | 27/27  (100.00) | 29/29  (100.00) | 56/56  (100.00) | 0.93 | 1.00 | 0.97 |
| **CINE_LVOT** |  | 13/13  (100.00) | 13/13  (100.00) |  | 9/13  (69.23) | 9/13  (69.23) |  | 9/13  (69.23) | 9/13  (69.23) | 1.00 | 0.69 | 0.82 |
| **CINE_RVOT** |  | 10/10  (100.00) | 10/10  (100.00) |  | 5/10  (50.00) | 5/10  (50.00) |  | 5/10  (50.00) | 5/10  (50.00) | 0.83 | 0.50 | 0.62 |
| **CINE_SAX** | 33/35  (94.29) | 30/32  (93.75) | 63/67  (94.03) | 34/35  (97.14) | 31/32  (96.88) | 65/67  (97.01) | 32/35  (91.43) | 30/32  (93.75) | 62/67  (92.54) | 0.69 | 0.93 | 0.79 |
| **FST2_2CH** | 3/3  (100.00) | 3/4  (75.00) | 6/7  (85.71) | 1/3  (33.33) | 3/4  (75.00) | 4/7  (57.14) | 1/3  (33.33) | 2/4  (50.00) | 3/7  (42.86) | 0.43 | 0.43 | 0.43 |
| **FST2_3CH** |  | 4/4  (100.00) | 4/4  (100.00) |  | 3/4  (75.00) | 3/4  (75.00) |  | 3/4  (75.00) | 3/4  (75.00) | 0.38 | 0.75 | 0.50 |
| **FST2_4CH** | 1/2  (50.00) | 4/4  (100.00) | 5/6  (83.33) | 2/2  (100.00) | 4/4  (100.00) | 6/6  (100.00) | 1/2  (50.00) | 4/4  (100.00) | 5/6  (83.33) | 0.83 | 0.83 | 0.83 |
| **FST2_SAX** | 4/4  (100.00) | 3/3  (100.00) | 7/7  (100.00) | 0/4  (0.00) | 1/3  (33.33) | 1/7  (14.29) | 0/4  (0.00) | 1/3  (33.33) | 1/7  (14.29) | 1.00 | 0.14 | 0.25 |
| **HASTE_AX** | 16/16  (100.00) | 59/59  (100.00) | 75/75  (100.00) | 16/16  (100.00) | 59/59  (100.00) | 75/75  (100.00) | 16/16  (100.00) | 59/59  (100.00) | 75/75  (100.00) | 0.97 | 1.00 | 0.99 |
| **MOLLI+_SAX** |  | 28/29  (96.55) | 28/29  (96.55) |  | 29/29  (100.00) | 29/29  (100.00) |  | 28/29  (96.55) | 28/29  (96.55) | 0.88 | 0.97 | 0.92 |
| **MOLLI-_SAX** |  | 25/30  (83.33) | 25/30  (83.33) |  | 30/30  (100.00) | 30/30  (100.00) |  | 25/30  (83.33) | 25/30  (83.33) | 1.00 | 0.83 | 0.91 |
| **PC_AORTA** |  | 13/13  (100.00) | 13/13  (100.00) |  | 13/13  (100.00) | 13/13  (100.00) |  | 13/13  (100.00) | 13/13  (100.00) | 0.54 | 1.00 | 0.70 |
| **PC_MPA** |  | 10/10  (100.00) | 10/10  (100.00) |  | 1/10  (10.00) | 1/10  (10.00) |  | 1/10  (10.00) | 1/10  (10.00) | 0.25 | 0.10 | 0.14 |
| **PERF_SAX** | 0/17  (0.00) |  | 0/17  (0.00) | 17/17  (100.00) |  | 17/17  (100.00) | 0/17  (0.00) |  | 0/17  (0.00) | 1.00 | 0.00 | 0.00 |
| **Scouts_MP** | 8/8  (100.00) | 89/90  (98.89) | 97/98  (98.98) | 8/8  (100.00) | 89/90  (98.89) | 97/98  (98.98) | 8/8  (100.00) | 89/90  (98.89) | 97/98  (98.98) | 1.00 | 0.99 | 0.99 |
| **TestPERF_SAX** | 4/15  (26.67) |  | 4/15  (26.67) | 15/15  (100.00) |  | 15/15  (100.00) | 4/15  (26.67) |  | 4/15  (26.67) | 1.00 | 0.27 | 0.42 |
| **TIscout_SAX** | 15/18  (83.33) | 54/55  (98.18) | 69/73  (94.52) | 18/18  (100.00) | 51/55  (92.73) | 69/73  (94.52) | 15/18  (83.33) | 51/55  (92.73) | 66/73  (90.41) | 0.89 | 0.90 | 0.90 |
| **WBLGE_2CH** | 33/34  (97.06) | 44/47  (93.62) | 77/81  (95.06) | 31/34  (91.18) | 41/47  (87.23) | 72/81  (88.89) | 30/34  (88.24) | 41/47  (87.23) | 71/81  (87.65) | 0.95 | 0.88 | 0.91 |
| **WBLGE_3CH** | 23/25  (92.00) | 37/37  (100.00) | 60/62  (96.77) | 20/25  (80.00) | 36/37  (97.30) | 56/62  (90.32) | 18/25  (72.00) | 36/37  (97.30) | 54/62  (87.10) | 0.92 | 0.87 | 0.89 |
| **WBLGE_4CH** | 28/30  (93.33) | 50/50  (100.00) | 78/80  (97.50) | 30/30  (100.00) | 50/50  (100.00) | 80/80  (100.00) | 28/30  (93.33) | 50/50  (100.00) | 78/80  (97.50) | 0.94 | 0.97 | 0.96 |
| **WBLGE_SAX** | 16/21  (76.19) | 54/64  (84.38) | 70/85  (82.35) | 20/21  (95.24) | 58/64  (90.63) | 78/85  (91.76) | 16/21  (76.19) | 50/64  (78.13) | 66/85  (77.65) | 0.87 | 0.78 | 0.82 |

bSSFP = balanced steady state free precession imaging

CH = chamber

LVOT = left ventricular outflow tract, perpendicular to 3-chamber plane

RVOT = right ventricular outflow tract (oblique sagittal plane)

EGE = early gadolinium enhanced images

FST2 = fat suppressed T2 weighted imaging

HASTE = half Fourier acquisition single shot turbo spin echo imaging

MOLLI+ = Modified Look Locker Inversion Recovery imaging post contrast

MOLLI- = native Modified Look Locker Inversion Recovery imaging

MPA = main pulmonary artery

SAX = short axis

T2BBMAPPING = T2 bright blood mapping

TI scout = inversion time scout imaging for late gadolinium enhanced imaging

WBLGE = white blood late gadolinium enhanced images, with nulling of normal myocardium

**Supplementary Figures**

**
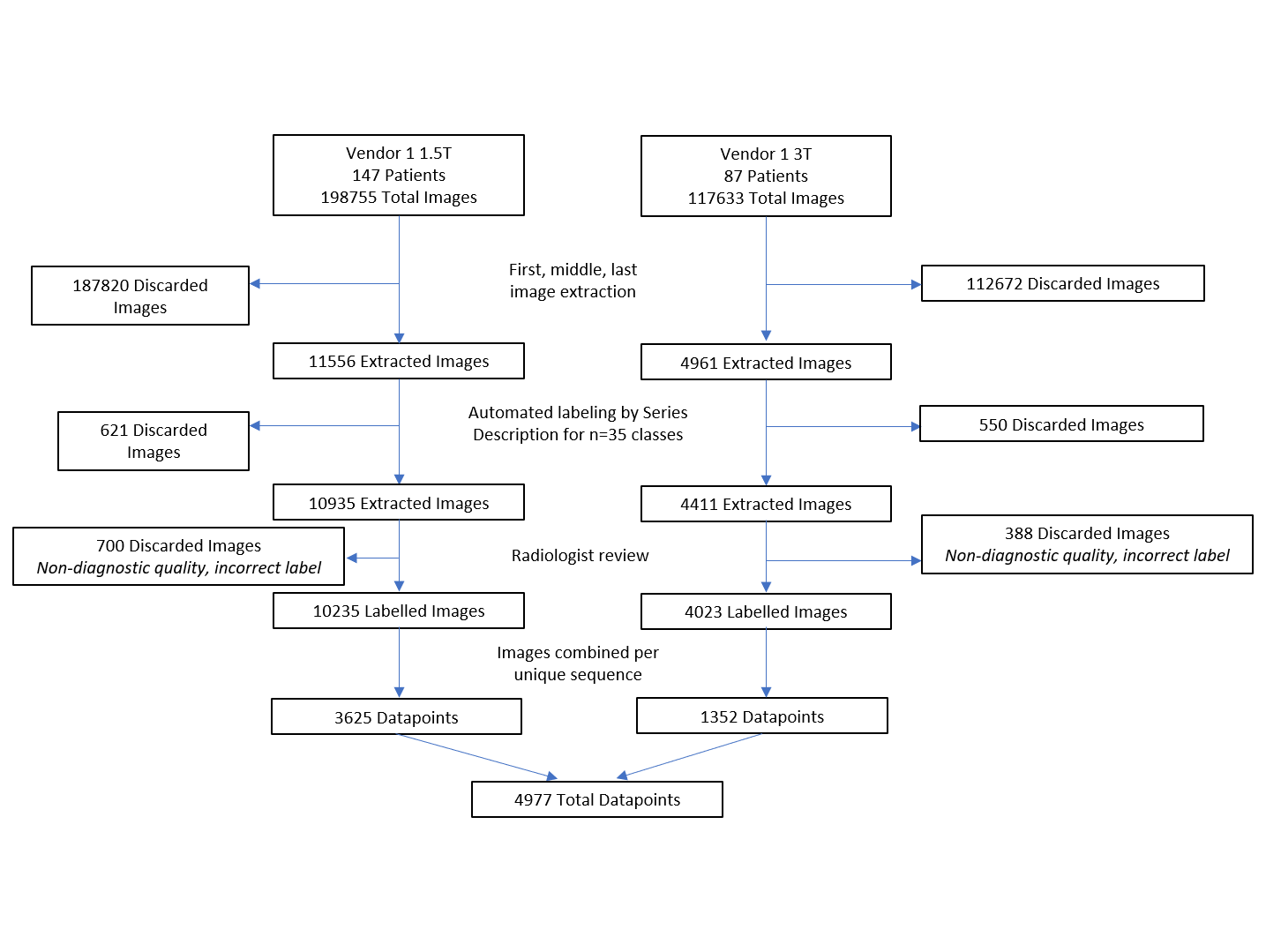
**

**Supplementary Figure 1a.** Data flow for Centre 1, Vendor 1

**
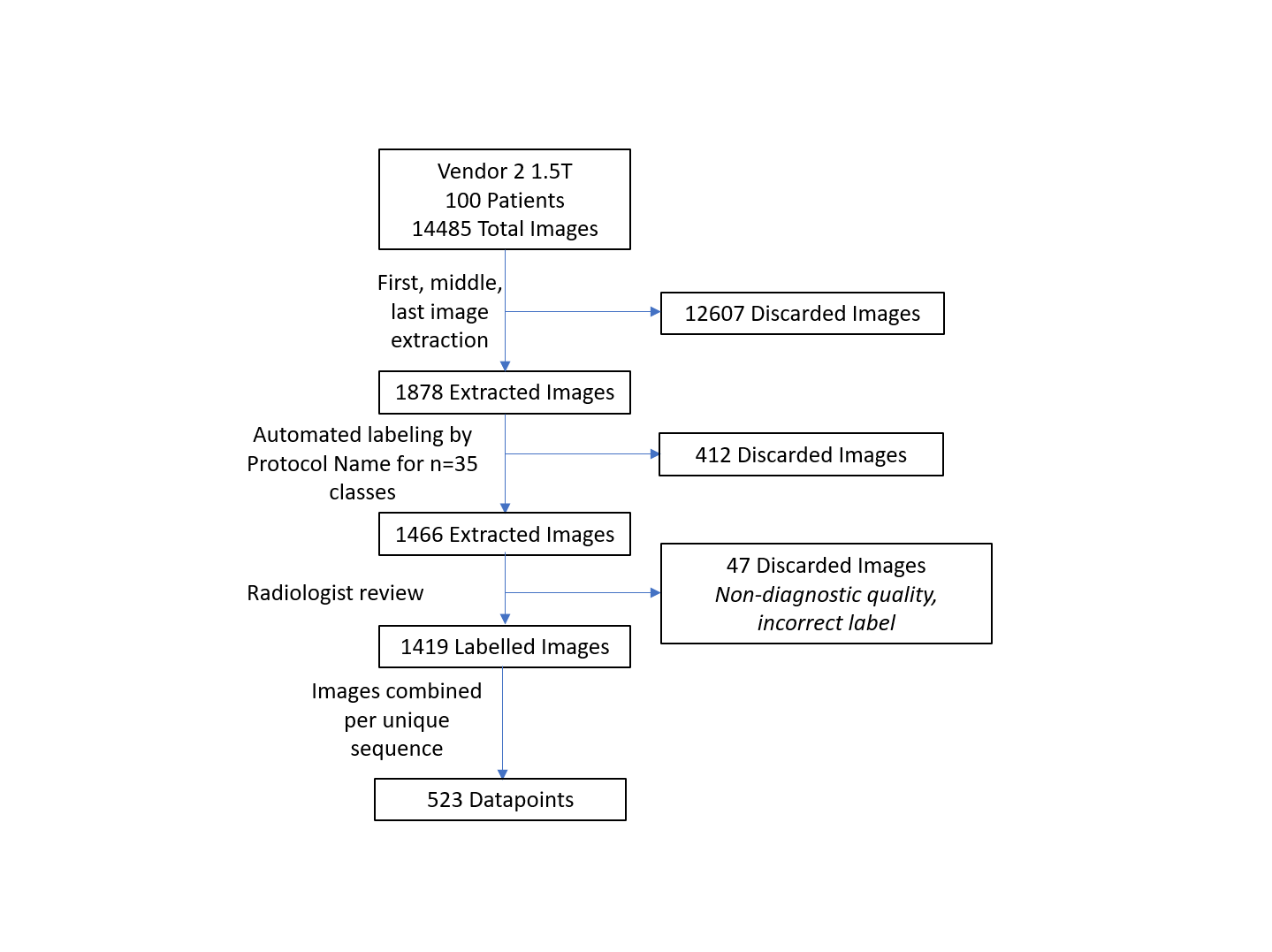
**

**Supplementary Figure 1b.** Data Flow for Centre 1, Vendor 2

**
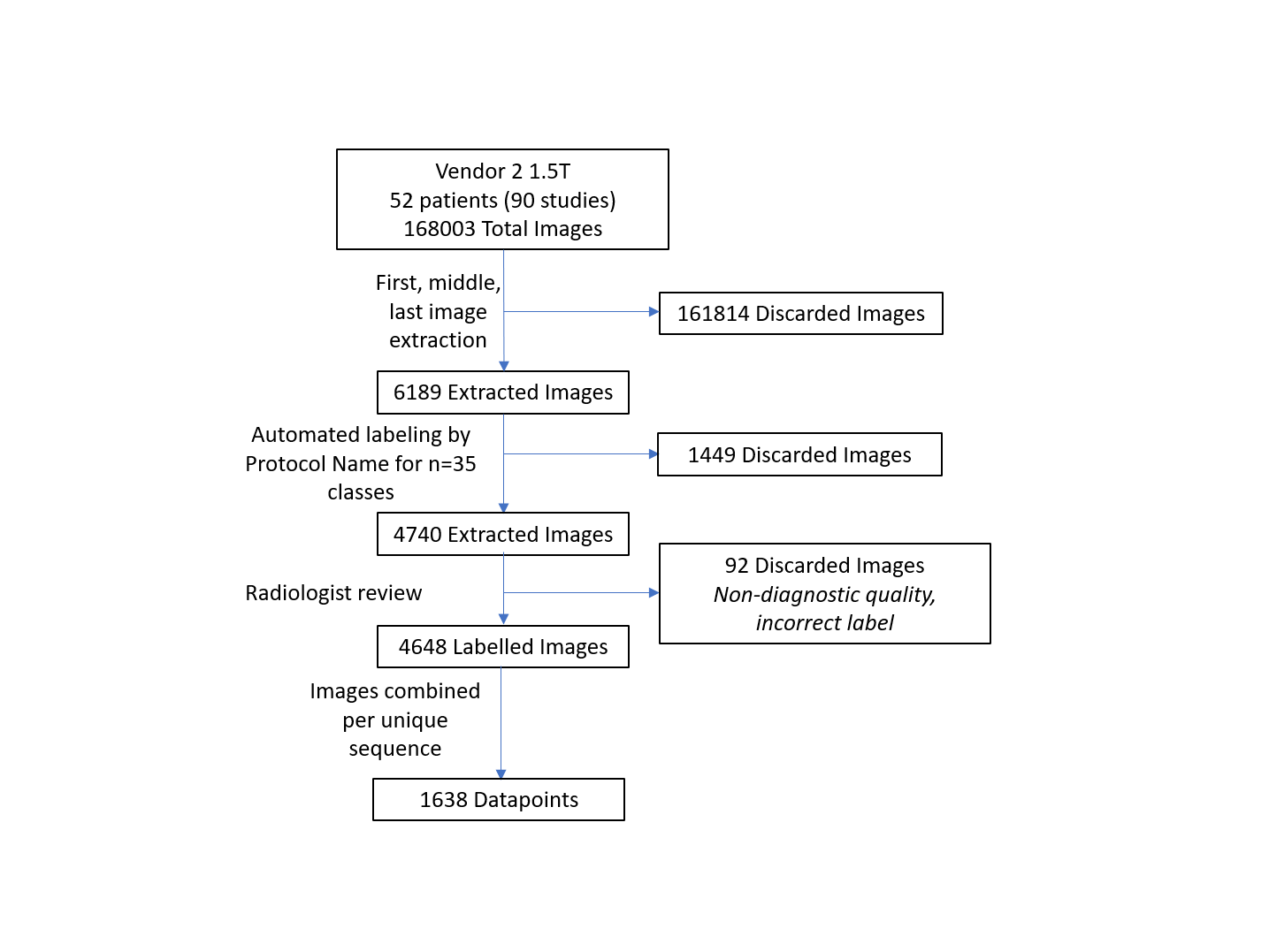
**

**Supplementary Figure 1c.** Data Flow for Centre 2, Vendor 2

**
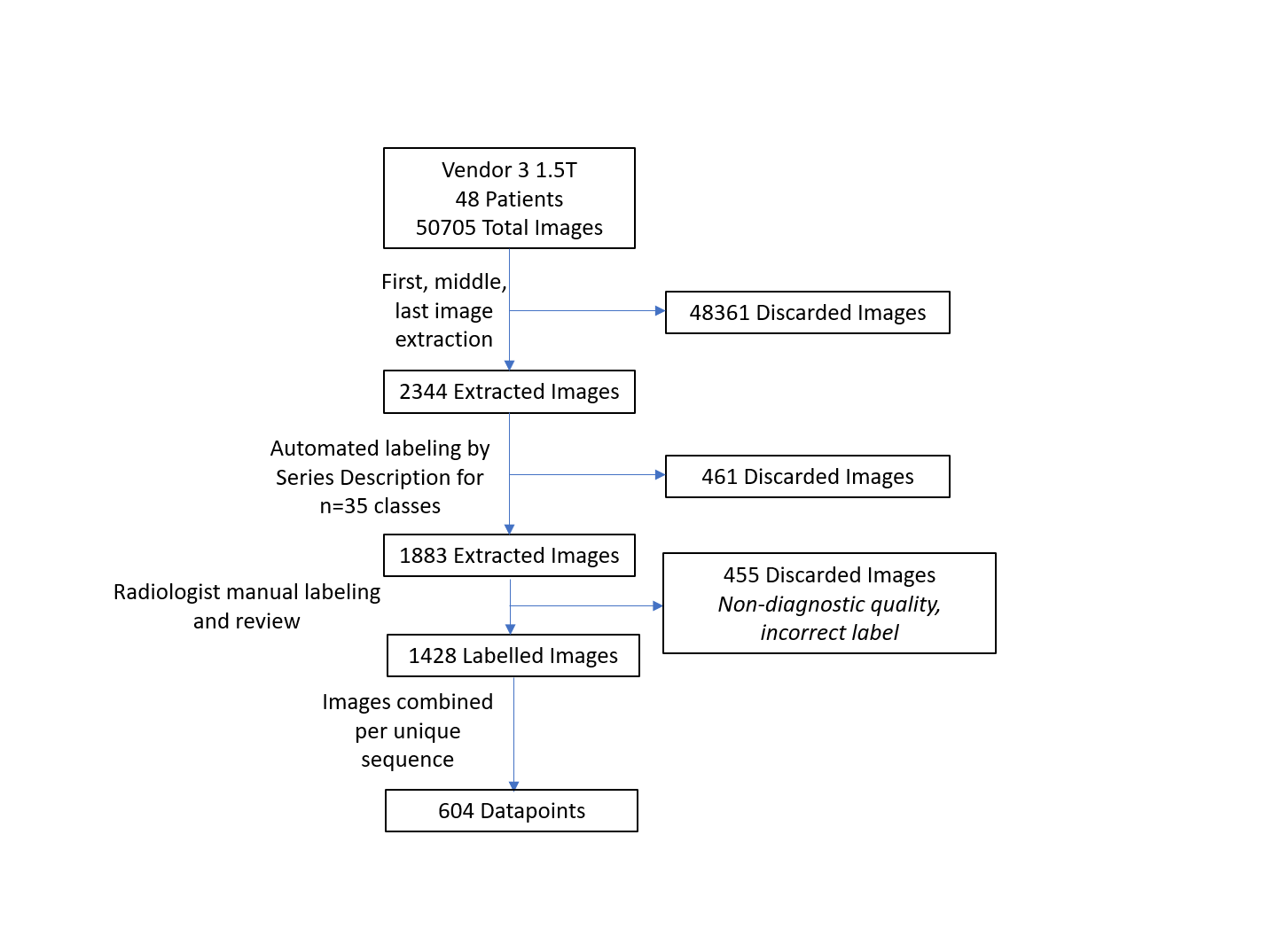
**

**Supplementary Figure 1d.** Data flow for Centre 3, Vendor 3

**
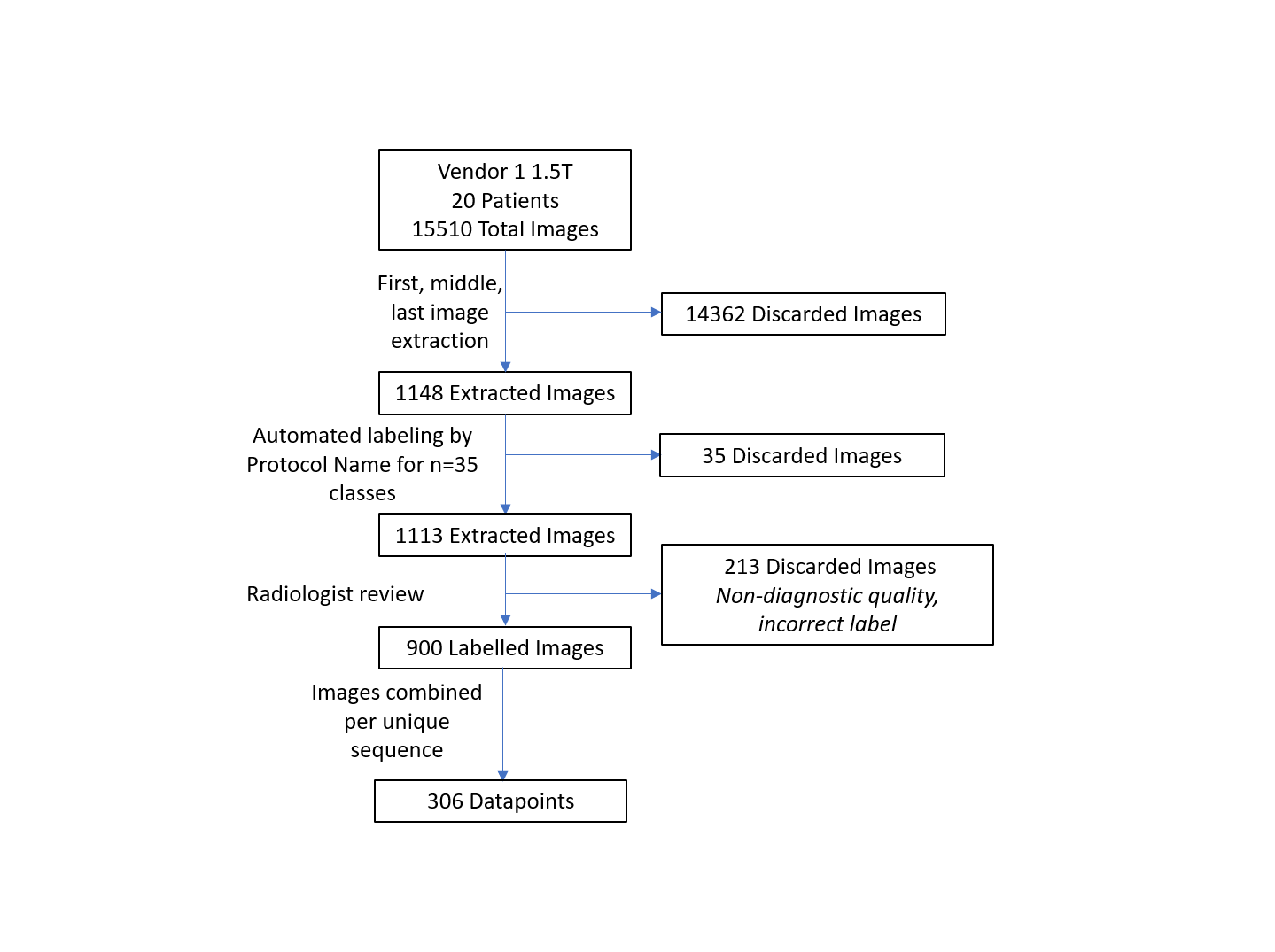
**

**Supplementary Figure 1e.** Data Flow for Centre 2, Vendor 1 (External Data)

**
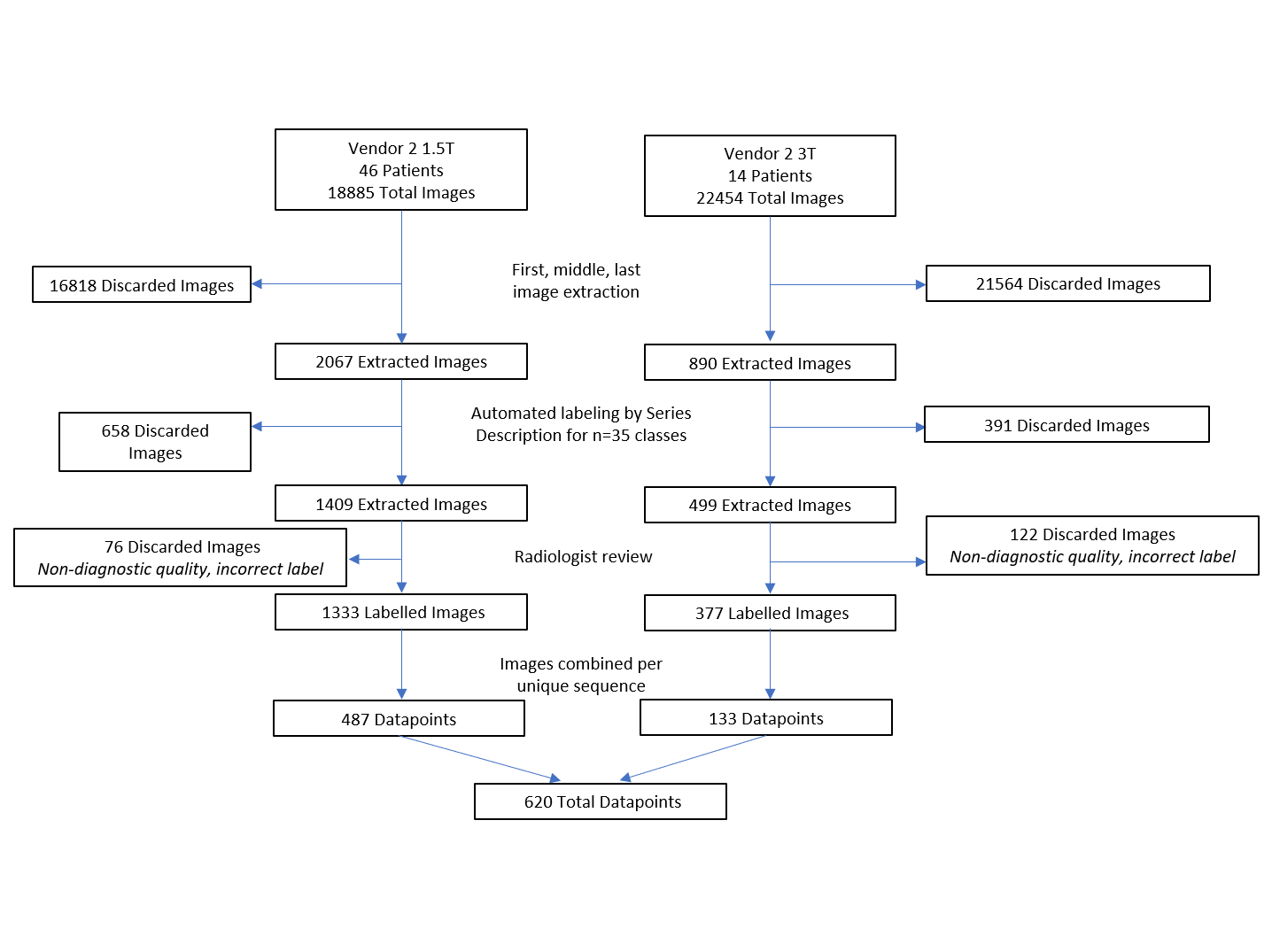
**

**Supplementary Figure 1f.** Data Flow for Centre 4, Vendor 2 (External Data)

**Supplementary Methods**

*Data Augmentation*

Random image augmentation was performed on the training dataset, employing alteration of Gaussian noise, image contrast, and composite affine and deformable geometric transformations, with extremes of augmentation reviewed by an expert radiologist to ensure augmented images remained recognisable by sequence type and imaging plane[1].

Specific augmentations performed:

A. Affine transformations:

1. Rotation: randomly selected between angles of -π /8 and +π/8 around the centre of the image
2. Anisotropic scaling: between 0.6 and 1.4x original image size in x and y dimensions
3. Translation: between -9% and 9% in x and y dimensions

B. B-spline transformation:

Using uniform randomly sampled displacements (between -14% and 14%) of a coarse 2D 4 x 4 B-spline grid.

C. Contrast

Scaling using a gamma transform with exponent between 0.9 and 1.1.

D. Image noise

Gaussian noise was added with a standard deviation between 0.01 and 0.05.

Order of image channels for each training datapoint was also randomized.

For all experiments, minority classes were oversampled for the training dataset[2], with different random augmentations applied to each new sample to avoid overfitting, such that the proportion of least to most common class representation was at least 1:4, empirically chosen to mitigate class imbalance whilst maintaining some real-world proportion of more versus less common sequences. For MVT and MVT_external_, oversampling of training data was also performed to balance classes across vendors, with the proportion of least common to most common vendor empirically selected to be at least 1:2.

*Hyperparameters*

The Adam optimizer was employed for training[3] (β_1_ = 0.9 and β_2_ = 0.999), with initial (minimum) and maximal learning rates determined with a learning rate finder[4], resulting in minimum learning rates of 2 x 10^-4^ (SVT, MVT_external_) and 1 x 10^-4^ (MVT) and a maximum learning rate of 0.01 for all experiments. A cyclical triangular learning rate was used with a full learning rate cycle completed every 60 epochs [4].

Training was performed with a batch size of 32, and batch normalization[5]. Spatial dropout was employed after each max pooling layer for convolutional layers, and dropout after the two fully connected layers preceding the output layer, with a dropout rate of 0.2[6].

*Training platform*

The Keras environment with a TensorFlow backend (v2.5.0) was used for model development, with a GeForce RTX 3090 GPU (Nvidia Corporation, Santa Clara, USA) used for training.

**References**

1. Eppenhof KAJ, Pluim JPW. (2019) Pulmonary CT Registration through Supervised Learning with Convolutional Neural Networks. IEEE Transactions on Medical Imaging 38(5): 1097-1105.

2. Buda M, Maki A, Mazurowski MA (2018) A systematic study of the class imbalance problem in convolutional neural networks. Neural Netw 106:249-259.

3. Kingma DP, Ba J (2015) Adam: a method for stochastic optimization. In: 3rd International Conference on Learning Representations, ArXiv:1412.6980.

4. Smith LN (2015) Cyclical Learning Rates for Training Neural Networks. ArXiv:1506.01186.

5. Ioffe S, Szegedy C. (2015) Batch normalization: accelerating deep network training by reducing internal covariate shift. Proceedings of the 32nd International Conference on International Conference on Machine Learning 37: 448–456. Lille, France.

6. Srivastava N, Hinton G, Krizhevsky A, Sutskever I, Salakhutdinov R (2014) Dropout: A Simple Way to Prevent Neural Networks from Overfitting. Journal of Machine Learning Research 15(56):1929-1958.
